# DNMT3b/OCT4 expression confers sorafenib resistance and poor prognosis of hepatocellular carcinoma through IL-6/STAT3 regulation

**DOI:** 10.1186/s13046-019-1442-2

**Published:** 2019-11-26

**Authors:** Ssu-Chuan Lai, Yu-Ting Su, Ching-Chi Chi, Yung-Che Kuo, Kam-Fai Lee, Yu-Chih Wu, Pei-Chi Lan, Muh-Hwa Yang, Te-Sheng Chang, Yen-Hua Huang

**Affiliations:** 1 Graduate Institute of Medical Sciences, College of Medicine, Taipei Medical University, Taipei, 11031 Taiwan; 2 Department of Biochemistry and Molecular Cell Biology, School of Medicine, College of Medicine, Taipei Medical University, Taipei, 11031 Taiwan; 3 TMU Research Center for Cell Therapy and Regeneration Medicine, Taipei Medical University, Taipei, 11031 Taiwan; 4 Department of Dermatology, Chang Gung Memorial Hospital, Linkou Taoyuan, 33305 Taiwan; 5 College of Medicine, Chang Gung University, Taoyuan, 33302 Taiwan; 6 Department of Pathology, Chang Gung Memorial Hospital, Chiayi, 61363 Taiwan; 7 School of Respiratory Therapy, College of Medicine, Taipei Medical University, Taipei, 11031 Taiwan; 8 Institute of Clinical Medicine, College of Medicine, National Yang Ming University, Taipei, 11221 Taiwan; 9 Division of Medical Oncology, Taipei Veterans General Hospital, Taipei, 11217 Taiwan; 10 School of Traditional Chinese Medicine, College of Medicine, Chang Gung University, Taoyuan, 33382 Taiwan; 11 Division of Internal Medicine, Department of Gastroenterology and Hepatology, Chang Gung Memorial Hospital, Chiayi, 61363 Taiwan; 12 International PhD Program for Cell Therapy and Regeneration Medicine, College of Medicine, Taipei Medical University, Taipei, 11031 Taiwan; 13 Center for Reproductive Medicine, Taipei Medical University Hospital, Taipei Medical University, Taipei, 11031 Taiwan; 14 Ph.D. Program for Translational Medicine, College of Medical Science and Technology, Taipei Medical University, Taipei, 11031 Taiwan; 15 Comprehensive Cancer Center of Taipei Medical University, Taipei, 11031 Taiwan

**Keywords:** Interleukin-6, Phospho-STAT3, OCT4, DNMT3b, Drug resistance, Hepatocellular carcinoma

## Abstract

**Background:**

The inflammatory cytokine interleukin-6 (IL-6) is critical for the expression of octamer-binding transcription factor 4 (OCT4), which is highly associated with early tumor recurrence and poor prognosis of hepatocellular carcinomas (HCC). DNA methyltransferase (DNMT) family is closely linked with OCT4 expression and drug resistance. However, the underlying mechanism regarding the interplay between DNMTs and IL-6-induced OCT4 expression and the sorafenib resistance of HCC remains largely unclear.

**Methods:**

HCC tissue samples were used to examine the association between DNMTs/OCT4 expression levels and clinical prognosis. Serum levels of IL-6 were detected using ELISA assays (*n* = 144). Gain- and loss-of-function experiments were performed in cell lines and mouse xenograft models to determine the underlying mechanism in vitro and in vivo.

**Results:**

We demonstrate that levels of DNA methyltransferase 3 beta (DNMT3b) are significantly correlated with the OCT4 levels in HCC tissues (*n* = 144), and the OCT4 expression levels are positively associated with the serum IL-6 levels. Higher levels of IL-6, DNMT3b, or OCT4 predicted early HCC recurrence and poor prognosis. We show that IL-6/STAT3 activation increases DNMT3b/1 and OCT4 in HCC. Activated phospho-STAT3 (STAT-Y640F) significantly increased DNMT3b/OCT4, while dominant negative phospho-STAT3 (STAT-Y705F) was suppressive. Inhibiting DNMT3b with RNA interference or nanaomycin A (a selective DNMT3b inhibitor) effectively suppressed the IL-6 or STAT-Y640F-induced increase of DNMT3b-OCT4 and ALDH activity in vitro and in vivo. The fact that OCT4 regulates the DNMT1 expressions were further demonstrated either by OCT4 forced expression or DNMT1 silence. Additionally, the DNMT3b silencing reduced the OCT4 expression in sorafenib-resistant Hep3B cells with or without IL-6 treatment. Notably, targeting DNMT3b with nanaomycin A significantly increased the cell sensitivity to sorafenib, with a synergistic combination index (CI) in sorafenib-resistant Hep3B cells.

**Conclusions:**

The DNMT3b plays a critical role in the IL-6-mediated OCT4 expression and the drug sensitivity of sorafenib-resistant HCC. The p-STAT3 activation increases the DNMT3b/OCT4 which confers the tumor early recurrence and poor prognosis of HCC patients. Findings from this study highlight the significance of IL-6-DNMT3b–mediated OCT4 expressions in future therapeutic target for patients expressing cancer stemness-related properties or sorafenib resistance in HCC.

## Background

Hepatocellular carcinoma (HCC), the most common type of primary liver cancer, is one of the leading causes of cancer mortality worldwide [1]. Chronic viral hepatitis infection contributes to the majority of HCC cases worldwide [2]. Despite advances in prevention and treatment strategies for this fatal disease, the incidence and mortality rate of HCC remain high, particularly for the Asian population [3]. With more surveillance of high risk patients, more HCC cases may be detected at an early stage when they are amenable to curative therapies [4]. Surgical resection is the most widely accepted therapy with a curative intention for the early stage HCCs [5]. However, the high tumor recurrence rate--up to 70% 5 years after surgery--remains a significant challenge. For advanced HCC, sorafenib is the first approved targeted therapy that yields statistically significant but clinically limited benefits as indicated by a 2–3 months increase in overall survival compared with placebo [6, 7]. Drug resistance to sorafenib has been the major obstacle for its clinical application in HCC. Multiple mechanisms, including crosstalk involving phosphoinositide-3-kinase–protein kinase B (PI3K/Akt) and Janus kinase-signal transducers and activators of transcription (JAK-STAT) signaling and the fibroblast growth factor 19/fibroblast growth factor receptor 4 (FGF19/FGFR4) axis, are attributable to sorafenib resistance [8–11]. In addition, epigenetic regulations, including histone modification, aberrant expression of miRNAs, and DNA methylation, are frequently involved in cancer stemness properties and drug resistance.

The expression of stemness-related genes, including octamer-binding transcription factor 4 (OCT4), stimulates a small population of cancer cells to gain the stemness-like properties that are responsible for the initiation and maintenance of primary tumors [12], and for tumor recurrence in some cancers, including HCC [13, 14]. Our previous study demonstrated that expression of the pluripotent transcription factor OCT4 is correlated with HCC recurrence, and is upregulated by interleukin-6 (IL-6) in a STAT3-dependent manner [14]. IL-6 is a prototypical cytokine which plays a pro-tumourogenic function in inflammation-associated cancers [15]. Constitutive activation of IL-6 signaling has been shown to establish a microenvironment for tumor initiation and progression [16]. In addition, IL-6 has been correlated with tumor stage and cancer stem cell (CSC)-like properties in human HCC [17, 18].

OCT4 is expressed in embryonic stem cells, germ cells, and various human cancers [19–22]. It is known to be a master regulator of stem cell pluripotency and self-renewal [23]. Emerging evidence has shown a correlation between OCT4 expression and tumor initiation and CSC-like phenotypes in many cancers, including prostate cancer, melanoma, and HCC [24–26]. OCT4 is also considered to be one of the most critical epigenetic mediators [27, 28]. Several epigenetic modifications, including DNA methylation, chromatin remodeling and long non-coding RNAs, have been implicated in the regulation of OCT4 gene expression [21, 29, 30].

DNA methylation, the transfer of methyl groups to DNA molecules catalyzed by DNA methyltransferases (DNMTs), occurs almost exclusively in CpG islands in mammals. Non-CpG methylation occurs in embryonic stem cells and has recently been observed during human B cell differentiation [21, 31]. The DNMT family includes DNMT1, DNMT3a, and DNMT3b. DNMT1 has been shown to maintain methylation in somatic cells, and DNMT3a and DNMT3b are thought to be involved in de novo DNA methylation in embryonic stem cells and early embryos. It was recently found that DNMT1, DNMT3a, and DNMT3b are overexpressed in several human tumors, compared to levels in corresponding normal tissues [32–34]. Numerous studies have indicated that these DNMTs are associated with hepatocarcinogenesis [14, 33–38]. Chronic HBV infection has been shown to significantly stimulate the up-regulation of DNMT1, DNMT3a, and DNMT3b in tumors that were associated with HCC progression [36]. HBV infection has also been shown to be highly correlated with increases in IL-6 [39], and we have previously demonstrated that increases in IL-6 can stimulate OCT4 expression through IGF-1R in HBV-related HCC (HBV-HCC) [14]. However, the underlying mechanism linking IL-6, OCT4, and DNMTs with sorafenib resistance in HCC remains largely unknown.

This study demonstrates that activation of IL-6/STAT3 regulates OCT4 expression through DNMT3b, which is an indicator of early tumor recurrence and poor prognosis of HCC. DNMT3b inhibitor nanaomycin A showed a synergistic effect with sorafenib in the sorafenib resistant Hep3B cells. Findings from this study highlight the significance of IL-6-DNMT3b–mediated signaling as a potential therapeutic target for patients with HCC exhibiting cancer stemness-related properties and sorafenib resistance.

## Methods

### Cell lines

The Hep3B (HBV^+^ HBsAg^+^ human HCC, HB-8064) and HepG2 (HBV^−^ human hepatoblastoma, HB-8065) cells were purchased from the American Type Culture Collection (ATCC, Manassas, VA, USA). The Huh7 (HBV^−^ human HCC) cells were obtained from the Japanese Collection of Research Bioresources. The HepG2.2.15(HBV^+^ HBsAg^+^ human hepatoblastoma) cells were kindly provided by Dr. Jun-Jen Liu (School of Medical Laboratory Science and Biotechnology, College of MedicalScience and Technology, Taipei Medical University, Taipei, Taiwan). Mycoplasma-free cell lines were used in all of our experiments. The sorafenib-resistant cells (sorafenib-resistant Hep3B or HepG2.2.15 cells) were generated by treating the cells with low dose sorafenib (1 nM) in the beginning, followed by a 10% increase in the sorafenib concentration every week until the maximum tolerated doses had been reached [40]. All the cells were maintained in Dulbecco’s Modified Eagle Medium (DMEM, Gibco-BRL, Thermo Fisher Scientific Waltham, MA, USA) supplemented with 10% fetal bovine serum (FBS) and 3.7 g/L sodium bicarbonate (Sigma-Aldrich), penicillin streptomycin (PS) (Gibco), and 1% glutamax (Gibco). For the IL-6 treatment, cells in serum-free media were treated with IL-6 (50 ng/mL; Peprotech, USA).

### HCC tissues

Human frozen HCC tissues and sera were obtained from 144 patients who had received curative hepatectomy between 2004 and 2013 at Chang Gung Memorial Hospital, Chiayi, Taiwan (Additional file 1: Figure S1). This study was approved by the Institutional Review Board of Chang Gung Medical Foundation (Approval number: 101–3575B).

### Serum IL-6 ELISA assay

Concentrations of serum IL-6 from the HCC patients (*n* = 144) were determined using an ELISA assay for human IL-6 (Bio Legend, San Diego, CA, USA) according to the manufacturer’s instructions.

### RNA isolation and real-time reverse-transcription polymerase chain reaction

Cell lines and frozen HCC tissues were subjected to total RNA isolation and quantitative real-time reverse-transcription polymerase chain reaction (RT-qPCR). For cell lines, total RNA was extracted with the Easy Pure Total RNA Spin Kit (Bioman, Taipei, Taiwan) according to the manufacturer’s instructions. Frozen tissues were homogenized in liquid N_2_ and lysed in RNA extraction buffer. The cDNA synthesis was performed using random primers (Invitrogen, Carlsbad, CA, USA) and MMLV reverse transcriptase (Invitrogen) according to the manufacturer’s instructions. The RT-qPCR amplification was performed with the LightCycler® 480 SYBR Green I Master (Hoffman-La Roche, Basal, Switzerland). The primer sequences, annealing temperatures, and PCR cycling conditions are described in Additional file 1: Table S1. The beta-2 M was used as an internal control.

### Immunoblotting and antibodies

Human HCC cell lines were extracted using lysis buffer containing 1% TritonX-100, 150 mM NaCl, 1 mM EDTA and 10 mM Tris-HCl (pH 7.5) with a protease inhibitor cocktail (Roche Diagnostics, NA, USA). Protein concentration was measured by the bicinchoninic acid (BCA) assay (Pierce, Rockford IL, USA). Equal amounts of protein were separated through 8% or 10% SDS-PAGE, transferred to a PVDF membrane, and subsequently probed using primary antibodies (Additional file 1: Table S2) at 4 °C overnight. Horseradish peroxidase (HRP)-conjugated secondary antibodies (Jackson ImmunoResearch, West Grove, PA, USA) were used and the immunoreactive bands were visualized using the enhanced chemiluminescence system (Amersham Pharmacia Biotech, Buckinghamshire, UK).

### Short hairpin RNA and plasmids

The packaging pCMVΔR8.91 plasmid and the envelope VSV-G pMD.G plasmid were co-transfected with shDNMT3b#1 (TRCN0000035686, Taiwan RNAi consortium, Taipei, Taiwan), shDNMT3b#2 (TRCN0000035685), shDNMT1#1 (TRCN0000232749), shDNMT1#2 (TRCN0000021893), or shCtrl. (TRCN0000072260) plasmids into HEK293T cells using Turbofect transfection reagent according to the manufacturer’s instructions (Thermo Fisher Scientific, Waltham, MA, USA). STAT3-WT, STAT3-Y640F and STAT3-Y705F plasmids were kindly provide by Prof. Muh-Hwa Yang (National Yang-Ming University, Taipei, Taiwan). The pMXs-OCT4 plasmids were provided by Dr. Hung-Chih Kuo (Academia Sinica, Taipei, Taiwan).

### Aldehyde dehydrogenase (ALDH) activity assay

ALDH enzymatic activity was measured using an ALDEFLUOR™ assay kit (Stemcell Tech, Grenoble, France) and a fluorescence-activated cell sorting Calibur system (BD Biosciences, San Jose, CA, USA) according to the manufacturer’s manual instructions. Briefly, the cells were harvested and re-suspended in ALDEFLUOR™ assay buffer containing an ALDH substrates (1 μM per 1 × 10^6^ cells) at 37 °C for 60 min. For a negative control in each experiment, a sample of cells was incubated, under identical experimental conditions, with 50 mM of the specific ALDH inhibitor diethylaminobenzaldehyde (DEAB).

### Tumor xenograft mouse model

Female 8-week-old athymic nude mice (BALB/cAnN. Cg-Foxnlnu/CrlNarl) were obtained from the National Laboratory Animal Center and National Applied Research Laboratories (Taipei, Taiwan). The animal study protocol was approved by the Institutional Animal Care and Use Committee/Panel at Taipei Medical University, Taipei, Taiwan.

The mice were subcutaneously inoculated with Hep3B (5 × 10^6^ cells) in the left flank, then injected with PBS or IL-6 (200 ng) at the implantation site every 3 days. Tumor volumes, calculated as 0.5 × length × width^2^ (mm^3^), were measured at one-week intervals over 56 days. The tumor tissues were collected at week eight post-implantation.

For nanaomycin A experiments, female eight-week-old NOD-SCID mice (National Laboratory Animal center) were subcutaneously inoculated with Hep3B (5 × 10^6^ cells) in the left flank, and locally injected with IL-6 (200 ng) at 3 day internals over 12 weeks. The mice were then divided into three groups: control DMSO group (*n* = 6), low dose nanaomycin A (2 μM, *n* = 6), and high dose nanaomycin A (20 μM, *n* = 6). The mice were treated with either DMSO or nanaomycin A every two days for a two week period. All the mice were sacrificed at week 14 and analyzed by immunohistochemical staining assays against the DNMTs and OCT4.

### Immunohistochemical staining

Matched pairs of paraffin-embedded HCC samples and adjacent liver tissues from patients receiving hepatectomy for HCC were used to construct the tissue microarray [41]. To produce the TMA block, tissue cylinders (1.5 mm in diameter) were punched from the region of the donor block and were transferred to an 18 × 30 mm paraffin block with the use of an automatic tissue arrayer instrument (Autotiss 1000, Ever BioTechnology, Canada). Formalin-fixed, paraffin-embedded human TMA blocks or mice tissue blocks were cut into 5-μm-thick continuous sections and mounted on poly-L-lysine-coated glass slides. After deparaffinization using xylene, slides were rehydrated in graded alcohol washes, washed in tap water and heated in 0.01 M citric buffer (pH 6.0) for 10 min with autoclaving. After cooling, the slides were treated with 3% H_2_O_2_ in phosphate buffered saline (PBS) at room temperature for 30 min, washed with PBS, blocked with 0.5% triton X and 5% normal horse serum in PBS and then incubated with primary antibodies (Additional file 1: Table S2) at 4 °C overnight, followed by incubation with horseradish peroxidase (HRP)-conjugated secondary antibodies and detected using 3,3′-diaminobenzidine tetrachloride (DAB) as the chromogen (DakoCytomation, Carpentaria, CA, USA). The immune-stained sections were counterstained with hematoxylin, dehydrated and mounted. Sections were then quantitatively analyzed using the Image Scope software (Aperio Technologies, Vista, CA, USA). The positive staining intensities of p-STAT3, DNMT1, DNMT3a, DNMT3b, and OCT have been presented as the total intensity per total pixels. Three randomly selected high-power fields were analyzed for each tumor section.

### Transwell assay

Transwell assays were performed using 8-μm pore transwell chambers in 24-well plates (Corning Costar, Cambridge, MA, USA). The upper chambers were seeded with 1 × 10^5^ Hep3B cells in 100 uL of the serum-free DMEM/F12 medium. The lower chambers were filled with 800 uL of the DMEM/F12 medium containing 10% FBS. Subsequently, the cells were incubated at 37 °C in a 5% CO_2_ humidified atmosphere for 24 h. The cells were treated with nanaomycin A in dose dependently. 48 h later, cells in the top side of the chamber was removed and cells in the bottom side of the chambers were fixed in 95% alcohol for 30 min, and then stained with crystal violet. The number of cells per three randomly selected fields was counted under the microscope (Olympus, Tokyo, Japan).

### Cell viability assay

For the proliferation assay, the naïve or sorafenib-resistant Hep3B/HepG2.2.15 cells were seeded in 96-well plates at 10^4^ cells/well and incubated at 37 °C in 5% CO_2_ for 48 h. For the drug sensitivity assay, the cells were seeded for 24 h and treated with various concentrations of sorafenib (#8705 Cell Signaling), or liposomal doxorubicin (Lipo-DOX, TTY Biopharm Company Limited, Taiwan), and these cells were then incubated at 37 °C in 5% CO_2_ for 48 h. Thereafter, a WST-1 assay (Roche) was used to detect cell proliferation according to the manufacturer’s instructions. Three experiments were performed for each experimental condition. Cell viability is expressed as the percentage of non-treated cells.

### The drug combination index (CI)

The effects of drug combinations were evaluated using Chou–Talalay median effect analysis. Cells were treated with single drug alone or in combination. The cell proliferation was measured using WST-1 assay, and the drug combination index (CI) was examined by the Chou–Talalay method. The CI values of < 0.9 were considered as evidence of synergy; 0.9–1.1 of additive effects; and CI > 1.1 of antagonism.

### Statistical analysis

Data are presented as mean ± standard deviation (SD) or median (standard error of mean [SEM]), as appropriate. The statistical differences in the means were assessed using the unpaired student’s *t* test and one-way analysis of variance [18], or Spear-man’s correlation analysis (GraphPad Software, La Jolla, CA, USA) for all cases.

## Results

### The positive correlation between serum IL-6 levels, DNMT3b/1 expression, and OCT4 expression with poor prognosis of human HCC

**Fig. 1:**
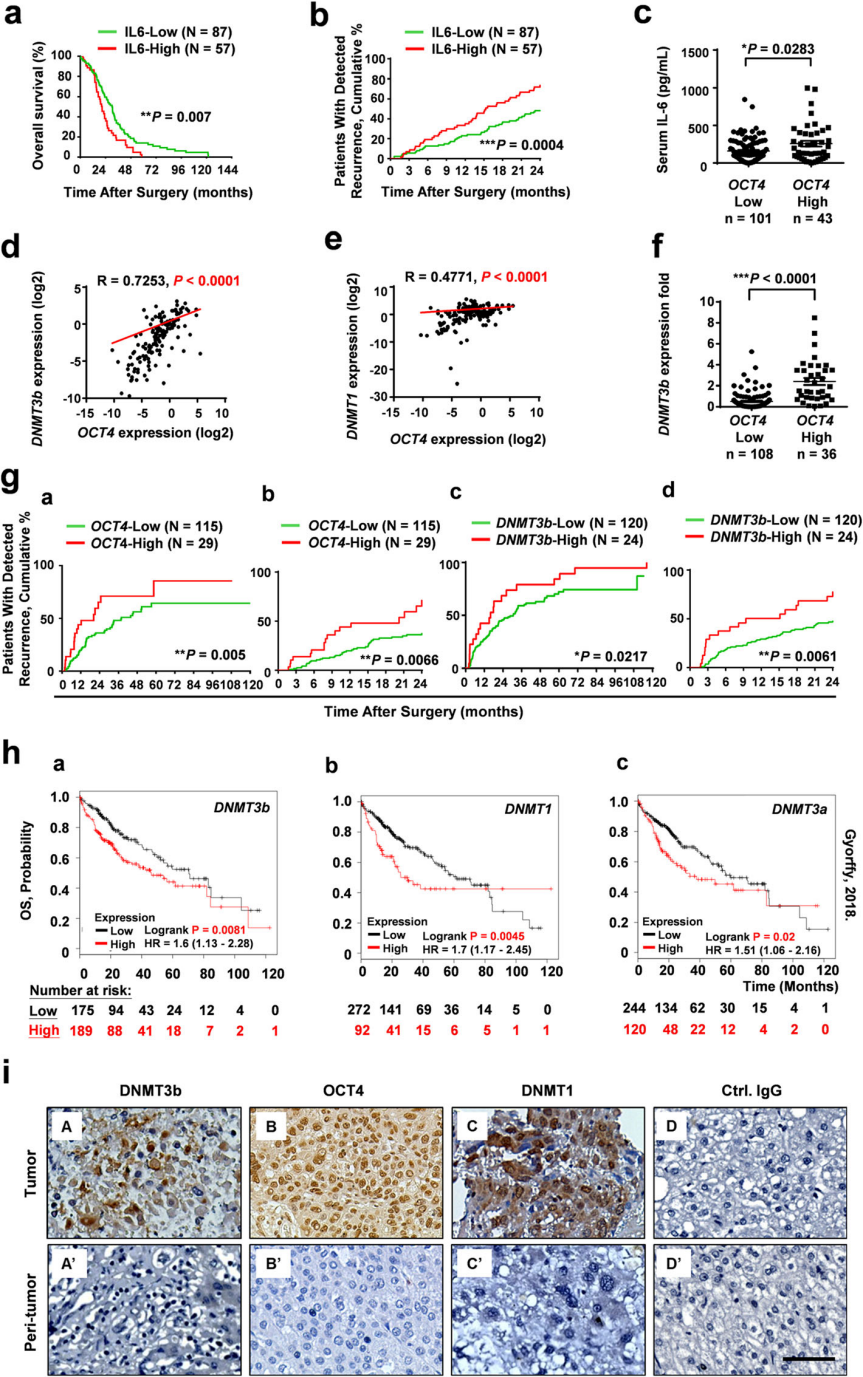
Correlation between serum IL-6 and tissue DNMT3b/OCT4 with the patient prognosis of human HCC. The overall survival (OS) (**a**) and early tumor recurrence (within 24 months) (**b**) of patients after HCC resection based on high or low serum IL-6 level by Kaplan-Meier analysis (*n* = 144, cutoff for high IL-6 concentration was 150 pg/mL). After normalization with the corresponding peritumor (PT) tissue sample, the expression levels (either high [T/PT ≧ 2 or low [T/PT < 2]) of *OCT4* were assessed. **c** The differences in serum levels of IL-6 between HCC patients with low OCT4 expression (T/PT < 2-fold; *n* = 101) and high OCT4 expression (T/PT ≥ 2-fold; *n* = 43) are shown. Positive correlations by Spearman analysis between expression levels of *OCT4* with *DNMT3b* (*R* = 0.7253) (**d**) and *DNMT1* (*R* = 0.4771) (**e**) in HCC tissues. *n* = 144. The differences in *DNMT3b* between HCC patients with low OCT4 expression (T/PT < 2-fold; *n* = 108) and high OCT4 expression (T/PT ≥ 2-fold; *n* = 36) (**f**) Statistical significance was assessed by the Mann–Whitney *U* test. (*P < 0.05; ***P < 0.001). **g** The Kaplan-Meier curves of tumor recurrence (120 months) or early recurrence (24 months) in relation to the transcriptional levels of *OCT4* (*n* = 144), *DNMT3b* (*n* = 144) in human HCC tissue (*P < 0.05, **P < 0.01). **h** The OS analysis of DNMT expression in HCC using The Cancer Genome Atlas (TCGA) dataset by Kaplan-Meier analysis (*n* = 364). The top tertile was defined as the high DNMTs expression cohort and the remaining patients were defined as the low DNMTs expression cohort. **i** The expression and localization of DNMT3b, OCT4, and DNMT1 in HCC tissues by immuno-histochemical staining. (Bar, 100 μm)

IL-6 has been associated with the expression of genes that contribute to stemness properties in HCC [13–15]. IL-6 has also been shown to upregulate DNA methyltransferase in several cancers [42–44]. To investigate the associations between serum IL-6 levels and *OCT4* and *DNMT* mRNA in human HCC tissues, serum IL-6 levels from 144 HCC patients were compared with *OCT4* and *DNMT* mRNA levels from paired frozen tumor tissue (T) and adjacent peritumor tissue (PT) samples (Table 1 and Additional file 1: Figure S1) using ELISA and real-time qRT-PCR. The expression levels (either high [T/PT ≧ 2] or low [T/PT < 2]) of *OCT4*, *DNMT3b,* and *DNMT1* were assessed. As shown in Fig. 1, we found that patients with high serum IL-6 levels showed a poorer overall survival (OS) compared with patients with low IL-6 levels (Fig. 1a, *P* = 0.007), and had more early tumor recurrence (Fig. 1b, *P* = 0.0004 for IL-6 and Table 1, *n* = 144). HCC patients who displayed higher levels of *OCT4* also had significantly higher levels of serum IL-6 (Fig. 1c). The patients who expressed both higher serum IL-6 and *OCT4* were more likely to have HBV-HCC than hepatitis C (HCV)-HCC (Additional file 1: Figure S2). We also observed significant positive correlations between *OCT4* expression levels and *DNMT3b* (Fig. 1d, *R* = 0.7253, *P* <  0.0001) and between *OCT4* and *DNMT1* (Fig. 1e, *R* = 0.4471, *P* <  0.0001). HCC patients who displayed higher levels of *OCT4* also had significantly higher levels of *DNMT3b* (Fig. 1f, *P* < 0.0001), and these patients with higher expression levels of *OCT4* (*P* = 0.005) and *DNMT3b* (*P* = 0.0217) had significantly higher rates of tumor recurrence (over 120 months), and of early recurrence (within 24 months) (Fig. 1g). In contrast, the correlation between *OCT4* and *DNMT3a* was relatively weak (Additional file 1: Figure S3).

**Table 1:** Variables associated with early tumor recurrence after hepatectomy for HCC (*N* = 144)

Variable	Recurrence^a^			
	Univariate analysis *P* value	Multivariate analysis		
		*P* value	Hazard ratio	95% confidence interval
Gender (male vs. female)	0.154	–	–	–
Age, years (< 55 vs. ≥ 55)	0.707	–	–	–
HBV (yes vs. no)	0.540	–	–	–
HCV (yes vs. no)	0.647	–	–	–
Bilirubin, mg/dL (< 1.2 vs. ≥ 1.2)	0.863	–	–	–
Albumin, g/dL (< 3.5 vs. ≥ 3.5)	0.252	–	–	–
ALT, U/L (< 35 vs. ≥ 35)	0.207	–	–	–
PT, INR (< 1.2 vs. ≥ 1.2)	0.075	0.104	2.827	0.808–9.885
ICG, retention rate (< 15% vs. ≥ 15%)	0.170	–	–	–
TNM stage (T1/2 vs. T3/4)	0.417	–	–	–
Multiple tumors (yes vs. no)	0.739	–	–	–
Child-Pugh class (A vs. B)	0.251	–	–	–
Complete tumor capsule (yes vs. no)	0.051	0.081	0.418	0.157–1.112
Microvascular invasion (yes vs. no)	**0.001**^**b**^	0.398	1.468	0.603–3.578
Macrovascular invasion (yes vs. no)	**< 0.001**^**b**^	**0.002**^**b**^	11.993	2.553–56.339
Cut margin free (yes vs. no)	0.873	–	–	–
Differentiation grade (1/2 vs. 3/4)	0.168	–	–	–
Tumor size, cm (< 3 vs. ≥ 3)	**0.039**^**b**^	0.554	0.788	0.359–1.733
Satellite nodules (yes vs. no)	**0.008**^**b**^	0.969	1.019	0.393–2.640
AFP, ng/mL (< 400 vs. ≥400)	0.625	–	–	–
Serum IL-6, ng/mL (< 150 vs. ≥ 150)	**0.023**^**b**^	0.088	1.896	0.910–3.949
Serum TGF-β (< 14,000 vs. ≥ 14,000)	0.682	–	–	–
*DNMT1* (< 2X vs. ≥ 2X)	**0.010**^**b**^	0.719	1.144	0.550–2.378
*DNMT3a* (< 2X vs. ≥ 2X)	0.305	0.239	1.632	0.722–3.689
*DNMT3b* (< 2X vs. ≥ 2X)	**0.037**^**b**^	0.199	1.779	0.739–4.285
*OCT4* (< 2X vs. ≥ 2X)	**0.026**^**b**^	0.085	1.990	0.909–4.357
*NANOG* (< 2X vs. ≥ 2X)	**0.004**^**b**^	**0.010**^**b**^	3.074	1.309–7.220
High expression of *OCT4* and *DNMT3b* (< 2X vs. ≥ 2X)	**0.001**^**b**^	0.395	1.484	0.597–3.685
High expression of *OCT4* and *DNMT1* (< 2X vs. ≥ 2X)	**0.013**^**b**^	0.226	1.754	0.705–4.364

Abbreviations: *AFP* serum α-fetoprotein, *ALT* alanine aminotransferase, *ICG* indocyanine green, *PT* prothrombin time, *INR* international normalized ratio, Tumor size, the largest one if multiple

^a^Time to early recurrence (less than 2 years)

^b^*P* < 0.05.

The clinical relevance of *OCT4*, *DNMT3b* and *DNMT1* expression levels in HCC prognosis was further examined using The Cancer Genome Atlas (TCGA) database and Kaplan–Meier analysis [45, 46]. As shown in Fig. 1h, Kaplan–Meier analysis showed that higher expression of *DNMT*s was associated with a trend for poor OS (*P* = 0.0081 for *DNMT3b*; *P* = 0.0045 for *DNMT1*; *P* = 0.02 for *DNMT3a*). These results were further supported by the other TCGA-PanCancer Atlas datasets from the cBioPortal for Cancer Genomics. Gene expression across these datasets revealed significantly higher expression levels of *DNMT3b, OCT4,* and *DNMT1* in the primary tumors compared with the normal tissues (Additional file 1: Figure S4a, b and c). In addition, there was a significant positive correlation between the gene expression levels of *OCT4* with *DNMT3b* (Additional file 1: Figure S4d, *R* = 0.2523, *P* < 0.0001) and *DNMT1* (Additional file 1: Figure S4e, *R* = 0.2121, *P* < 0.0001). Although the *DNMT3a* level in tumor tissues was higher than that of the normal tissues, there was no statistical significance between the expression levels of *OCT4* and *DNMT3a* in tumors (Additional file 1: Figure S4f and g).

The protein expressions of DNMT3b, OCT4, and DNMT1 in HCC tissues were also examined by immunohistochemical staining (Fig. 1i). Taken together, these results strongly suggest that the levels of IL-6, DNMT3b/1, and OCT4 are highly correlated and that they play a role in early tumor recurrence and poor prognosis of HCC patients.

### IL-6 activates the expression of DNMT3b, OCT4, and DNMT1 in Hep3B cells in vitro and in vivo

**Fig. 2:**
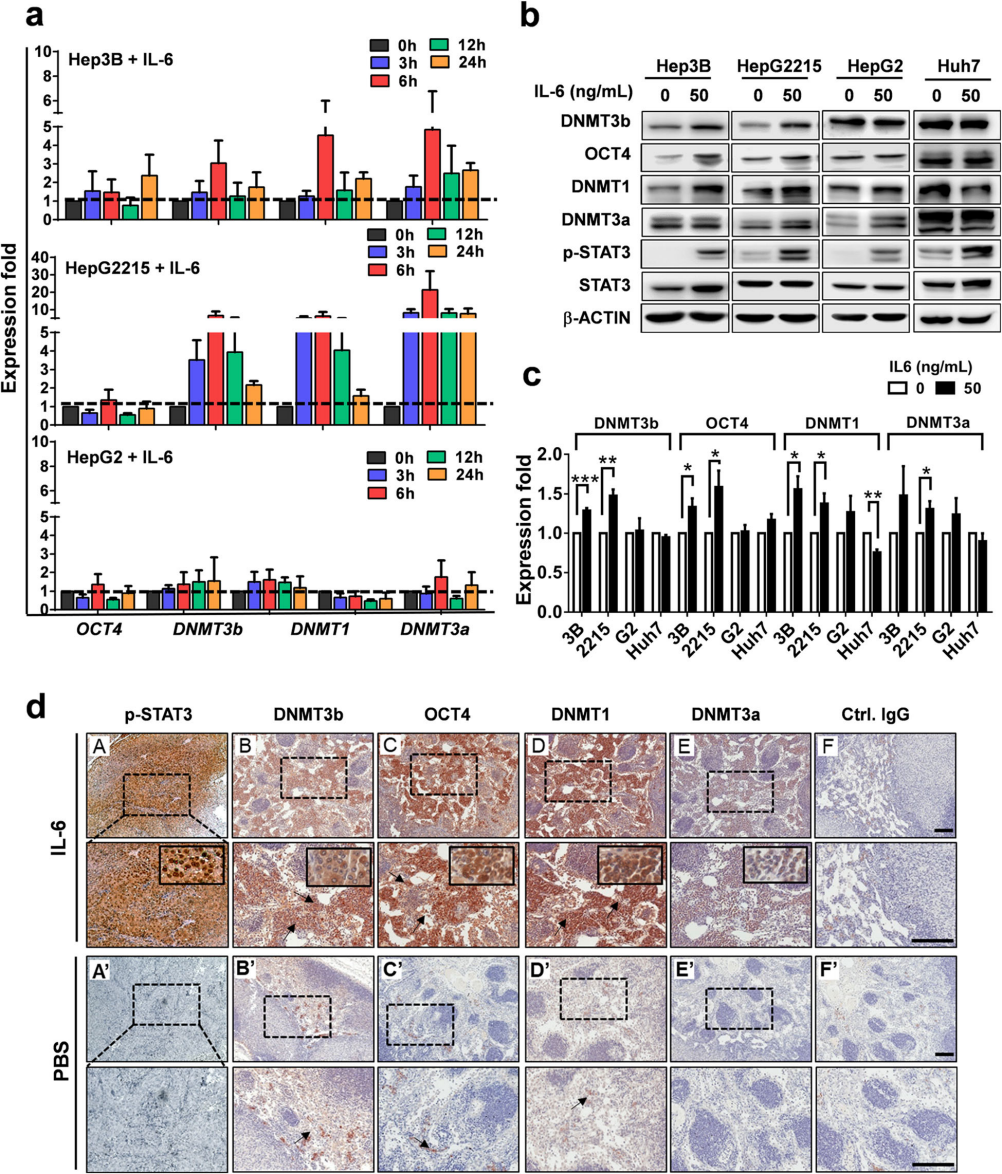
IL-6 activates the expression of DNMTs and OCT4 in vitro and in vivo*.* **a** The effect of IL-6 (50 ng/mL) on mRNA expression of *DNMTs* and *OCT4* in HBV^+^HBsAg^+^ HCCs (Hep3B and HepG2.2.15) and HBV^−^HBsAg^−^ HCCs (HepG2) under different incubation time assessed by real-time qPCR. **b** The effect of IL-6 (50 ng/mL) on the protein expression of *DNMTs* and *OCT4* in HBV^+^HBsAg^+^ HCCs (Hep3B and HepG2.2.15) and HBV^−^HBsAg^−^ HCCs (HepG2 and Huh7) by western blotting. The qualitative data is shown in (**c**). Three individual experiments were carried out for each experimental condition. *P < 0.05, **P < 0.01, ***P < 0.0001, student *t* test. **d** Xenografts of 5 × 10^6^ Hep3B cells in athymic nude mice with IL-6 (200 ng/mouse/3 d) treatment (*n* = 6). Immuno-histochemical staining for p-STAT3, DNMT3b, OCT4, DNMT1, DNMT3a, and the control IgG are shown (Bar, 100 μm)

HCC patients with virus infection have been shown to have high expression of IL-6 [14]. As we found a positive correlation between serum IL-6 levels and *OCT4* expression in HCC (Fig. 1c), we next examined the effect of IL-6 on expression levels of OCT4 and DNMTs in HCC cells. Human HCC cell lines that contain the HBV genome (Hep3B and HepG2.2.15) or do not contain the HBV genome (HepG2 and Huh7) were used in this experiment, and the mRNA levels of *DNMTs* and *OCT4* were detected using qPCR. As shown in Fig. 2a, IL-6 treatment significantly increased *OCT4* and *DNMTs* mRNA expression, particularly in HBV^+^HBsAg^+^ Hep3B and HepG2.2.15 cells. Western blotting results further demonstrated that IL-6 significantly increased the protein expression of DNMT3b, OCT4, and DNMT1 in HBV^+^HBsAg^+^ Hep3B and HepG2.2.15 cells, but not in HBV^−^HBsAg^−^ HepG2 and Huh7 cells (Fig. 2b). The quantitative analytic results are shown in Fig. 2c.

Further experiments using a xenograft animal model confirmed that IL-6/p-STAT3 activation increased the expression of DNMT3b, OCT4 and DNMT1, and, to a much lesser extent, of DNMT3a, in Hep3B-derived tumors in vivo (*n* = 12). When compared to the PBS control group, immunohistochemical staining showed that IL-6 treatment dramatically increased p-STAT3 protein levels, and markedly increased the expression of OCT4, DNMT3b, and DNMT1 in Hep3B tumors (Fig. 2d). These results demonstrate that IL-6/p-STAT3 activates the expression of DNMT3b, OCT4, and DNMT1 in Hep3B both in vitro and in vivo.

### P-STAT3 activation enhances the expression of DNMTs and OCT4 and increases ALDH activity in human Hep3B cells

**Fig. 3:**
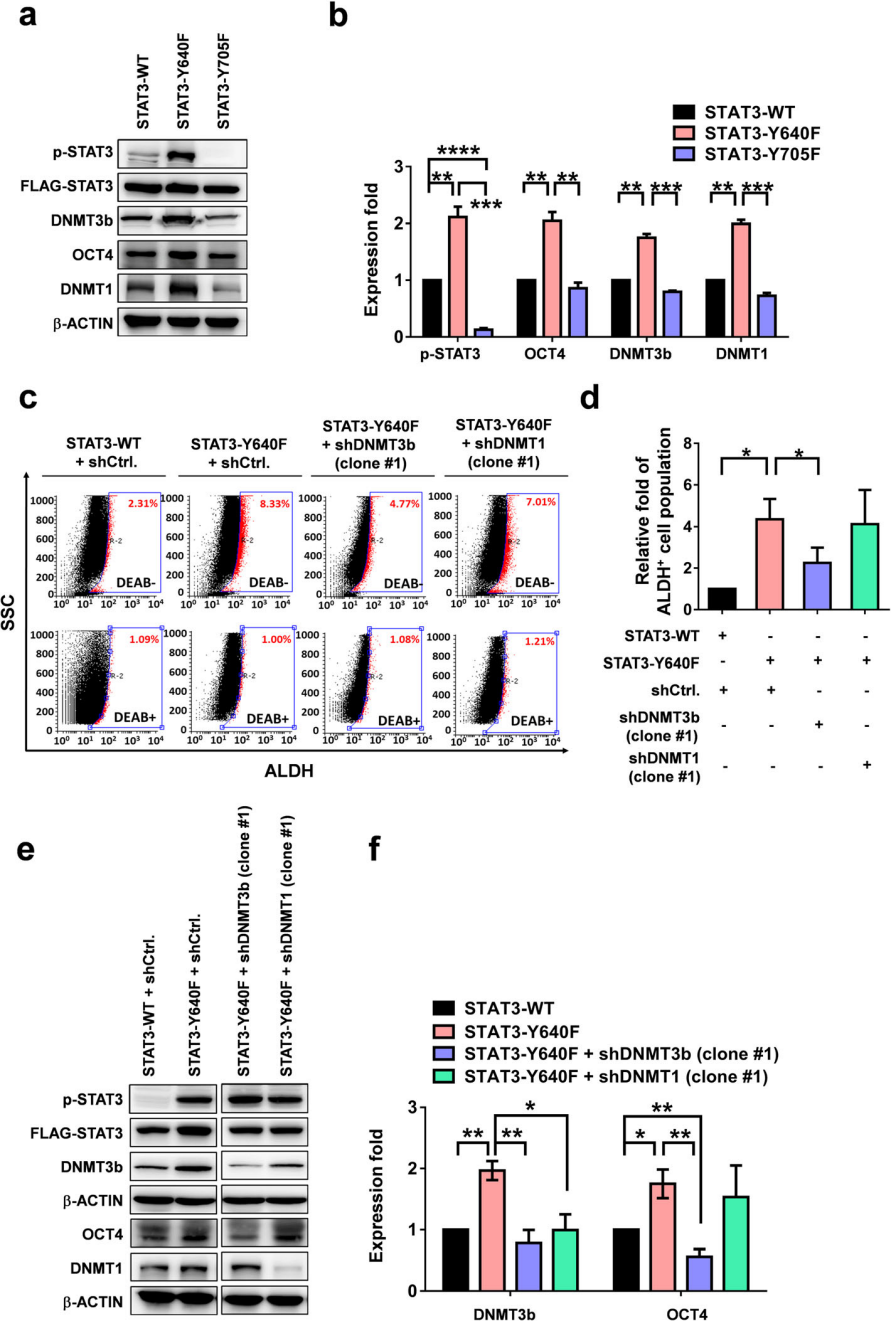
p-STAT3 activation enhances the expression of DNMTs/OCT4 and increases ALDH activity in Hep3B cells. The expression levels of DNMTs and OCT4 in Hep3B overexpressed with STAT3-WT, STAT3-Y640F (constitutively phosphorylated) or STAT3-Y705F (dominant negative). **a** Western blot analysis. **b** The quantitative data of (**a**). *P < 0.05, **P < 0.01, ***P < 0.001, ***P < 0.0001, student *t* test. **c** ALDH activity in Hep3B cells transfected with STAT3-WT, STAT3-Y640F or STAT3-Y705F. **d** The quantitative data of (**c**). *P < 0.05, student *t* test. ALDH, aldehyde dehydrogenase; DEAB, diethylaminobenzaldehyde; SSC, side scatter. **e** The protein levels of DNMT3b and OCT4 in STAT3-Y640F-Hep3B cells with or without RNA silencing of DNMT3b or DNMT1. shCtrl, control vector; shDNMT3b, DNMT3b silencing plasmid; and shDNMT1, DNMT1 silencing plasmid. **e** Western blot analysis. **f** The quantitative data of (**e**). *P < 0.05, **P < 0.01, student *t* test

To confirm the role of STAT3 signaling in the expression of DNMT and OCT4 in HCC cells, plasmids with constitutively activated p-STAT3 (STAT3-Y640F) and dominant negative p-STAT3 (DN STAT3-Y705F) were employed. We found that constitutively activated p-STAT3 (STAT3-Y640F) significantly increased the expressions of DNMT3b, OCT4, and DNMT1 in Hep3B cells compared with those in wild-type STAT3, whereas DN STAT3-Y705F exhibited a suppressive effect (Fig. 3a). The quantitation assay is presented in Fig. 3b.

To identify the specific DNMT that was responsible for the p-STAT3-induced stemness-related ALDH activity and the increased OCT4 expression, RNA interference experiments were conducted targeting DNMT3b (short hairpin [sh]DNMT3b) or DNMT1 (shDNMT1) in STAT3-Y640F Hep3B cells. Activated phospho-STAT3 (STAT3-Y640F) significantly enhanced the ALDH activity (Fig. 3c and d) in Hep3B cells. DNMT3b silencing effectively suppressed the STAT3-Y640F-induced ALDH activity (Fig. 3c and d) and the expressions of DNMT3b, OCT4, and DNMT1 (Fig. 3e and f). By contrast, DNMT1 knockdown exhibited less effect on the STAT3-Y640F-induced ALDH activity (Fig. 3c and d) and OCT4 expression (Fig. 3e and f). These results demonstrated that the epigenetic regulator DNMT3b regulated the p-STAT3-induced ALDH activity and OCT4 expression in Hep3B cells.

### IL-6 increases OCT4 expression through a DNMT3b-OCT4-DNMT1 axis

**Fig. 4:**
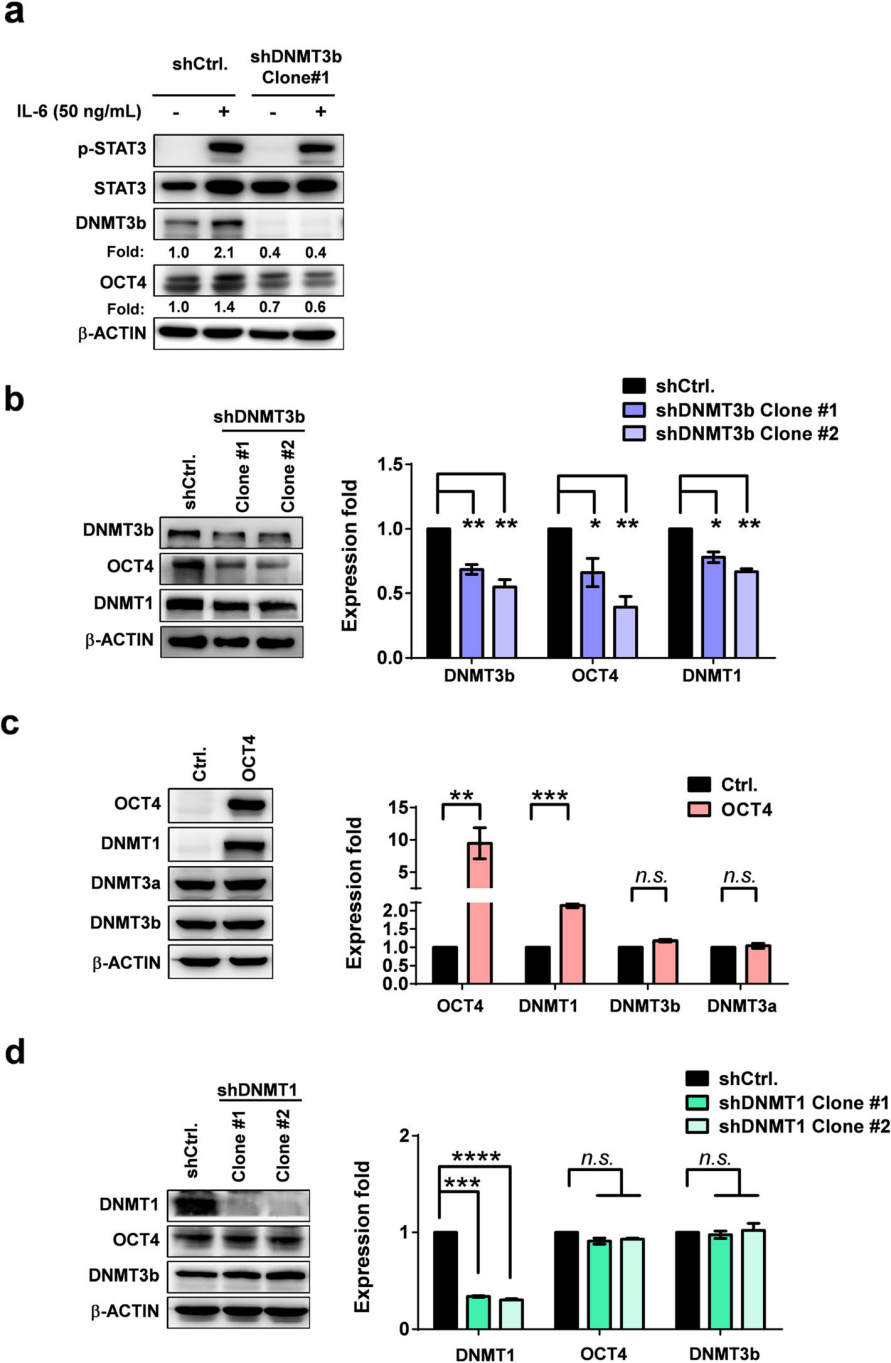
DNMT3b silencing reduces IL-6-induced OCT4 and forced OCT4 expression increases the DNMT1 in Hep3B cells. **a** Effect of DNMT3b silencing on the IL-6-induced OCT4 expression in Hep3B cells. **b** OCT4 and DNMT1 protein levels of the shCtrl.- or shDNMT3b Hep3B cells. **c** Effect of forced OCT4 expression on the protein levels of DNMT1, DNMT3a, and DNMT3b in Hep3B cells. **d** DNMT3b and OCT4 protein levels of the shCtrl.- or shDNMT1 Hep3B cells. Western blotting analysis. *P < 0.05, **P < 0.01, ***P < 0.001, ***P < 0.0001, student *t* test

To further examine whether the IL-6 regulates the OCT4 expression through a DNMT3b-OCT4-DNMT1 pathway, RNA interference experiments using shDNMT in Hep3B cells with and without IL-6 treatment were performed. The results revealed that IL-6 effectively increased the expression of DNMT3b and OCT4, and shDNMT3b (Clone #1) effectively suppressed the effect of IL-6 (Fig. 4a and Additional file 1: Figure S5). To further confirm the effect of DNMT3b on OCT4 expression, two effective shDNMT3b clones (Clone #1 and #2) were used in the experiments. We found that DNMT3b silencing significantly suppressed the OCT4 expression, and to a less extent, of DNMT1 (Fig. 4b). Forced expression of OCT4 significantly increased the cell viability (Additional file 1: Figure S6) and the DNMT1 expression, but had no effect on the DNMT3b or DNMT3a expression in Hep3B cells (Fig. 4c). By contrast, DNMT1 knockdown (shDNMT1 clone #1 and #2) did not exhibit any effect on the DNMT3b or OCT4 expression (Fig. 4d). These results demonstrated that IL-6 regulated the OCT4 expression through the DNMT3b-OCT4-DNMT1 axis in HCC.

### DNMT3b inhibitor suppresses IL-6-induced OCT4 expression and tumorigenicity in xenografted Hep3B tumors in NOD-SCID mice

**Fig. 5:**
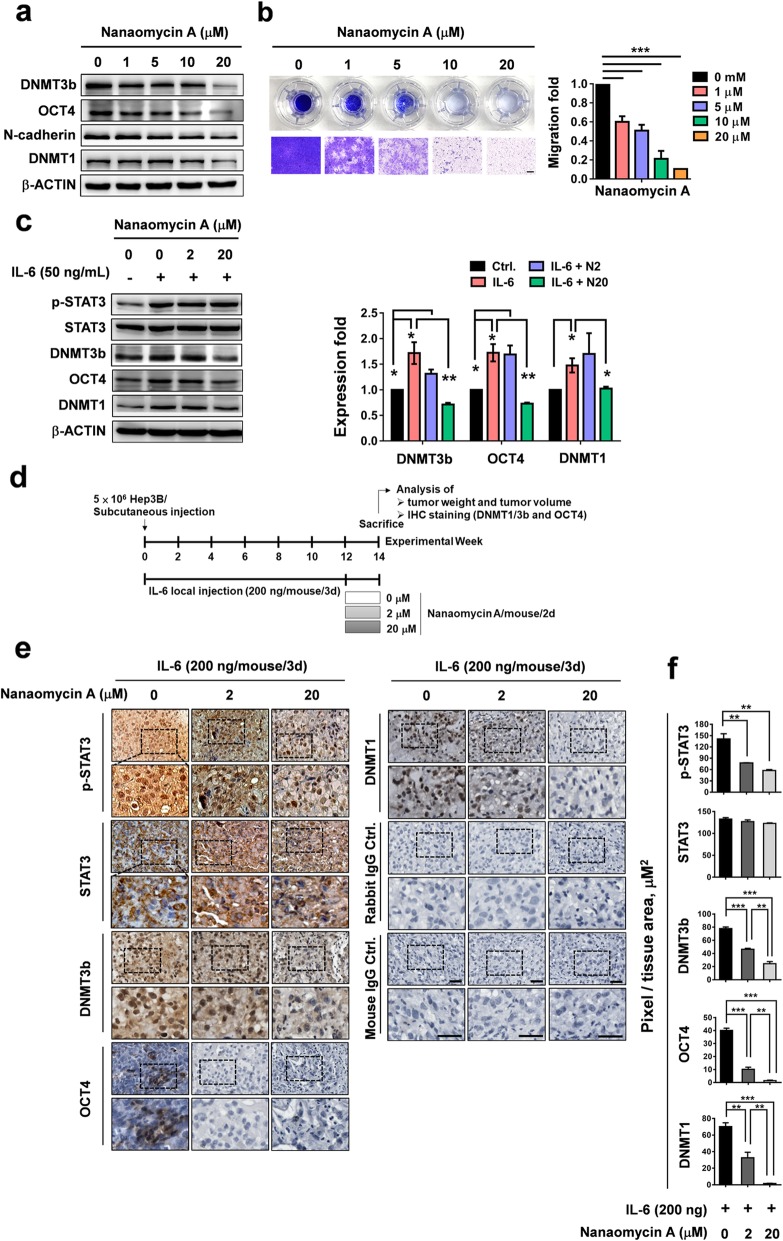
IL-6 increases OCT4 expression through DNMT3b regulation in vitro and in vivo*.* **a** Effect of nanaomycin A (a DNMT3b inhibitor) on protein expression of DNMT3b/1, OCT4, and N-cadherin in Hep3B cells. **b** The effect of nanaomycin A (0, 1, 5, 10, 20 μM) on the migration ability of Hep3B cells. Transwell assay. Bar = 100 um. **c** Effect of nanaomycin A on IL-6-induced protein expression of DNMT3b/1 and OCT4 in Hep3B cells. **d** The time course of IL-6/nanaomycin A-treated Hep3B animal models. *n* = 6 for each group. **e** Immunohistochemical analysis of the protein expressions of p-STAT3, STAT3, DNMT3b, OCT4, and DNMT1 following PBS, IL-6, and IL-6 plus nanaomycin A treatment (0, 2, 20 μM/mouse/2 days). Bar, 100 μm. **f** The quantitative data of (a). *P < 0.05, **P < 0.01, ***P < 0.001, student *t* test

To examine if targeting DNMT3b could effectively suppress OCT4 expression, the DNMT3b-selective inhibitor nanaomycin A [47], a unique quinone antibiotic isolated from *Streptomyce*s that induces genomic demethylation, was used. As shown in Fig. 5, we found that the nanaomycin A treatment decreased the basal protein levels of DNMT3b, OCT4, and N-cadherin (Fig. 5a) and the cell migration ability (Fig. 5b) of Hep3B cells in a dose-dependent manner. The low dose of nanaomycin (1 μM) can suppress the DNMT3b/OCT4 levels and cell migration ability more than 50%. IL-6 was able to induce protein expression of DNMT3b/1 and OCT4, and the nanaomycin A (20 μM) effectively suppressed the IL-6-induced expression of DNMT3b, OCT4, and DNMT1 (Fig. 5c).

The effect of nanaomycin A on DNMT expression in vivo was further examined using a xenograft NOD-SCID tumor model. NOD-SCID mice were subcutaneously inoculated with Hep3B, and then received local IL-6 treatment (200 ng/mouse/3 days) over a course of 12 weeks (Fig. 5d). After 12 weeks, the mice were divided into three groups based on nanaomycin A treatment. The immunostaining analysis showed that IL-6 effectively increased p-STAT3, STAT3, DNMT3b/1, and OCT4 expression in Hep3B-derived tumors, and that nanaomycin A significantly suppressed the effect of IL-6 in a dose-dependent fashion (Fig. 5e). The quantitative analytic results are shown in Fig. 5f. These results demonstrate that DNMT3b regulates the IL-6-induced expression of OCT4 in HCC both in vitro and in vivo.

### DNMT3b inhibitor increases the drug sensitivity of sorafenib-resistant HCC cells

**Fig. 6:**
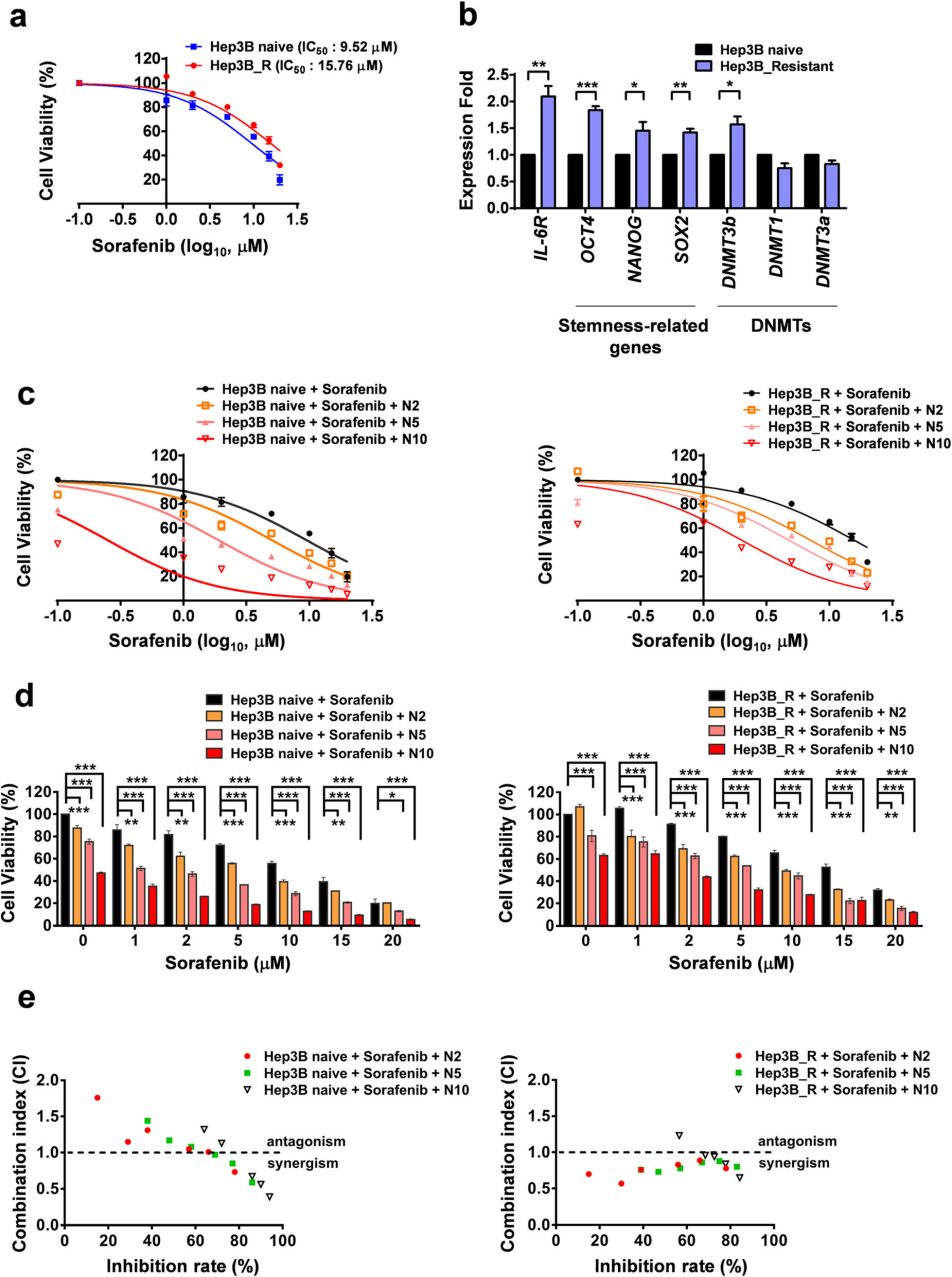
Combination of nanaomycin A increases the sorafenib susceptibility in Hep3B sorafenib resistant HCC cells. (**a**) The cell viability of Sorafenib-naïve Hep3B cells or sorafenib-resistant Hep3B cells under Sorafenib treatment (0, 1, 5, 10, 15 20 μM) (**b**) The mRNA levels of *IL-6R*, stemness-related genes, and *DNMTs* in Hep3B naïve/resistant cells were shown. Real-time Q-PCR assay. *P < 0.05, **P < 0.01, ***P < 0.001, student *t* test. (**c**, **d**) The cell viability of Hep3B naïve/resistant cells under the treatment of Sorafenib (1, 5, 10, 15 20 μM) with or without nanaomycin A (2, 5, 10 μM) for 48 h. The WST assay. *P < 0.05, **P < 0.01, ***P < 0.001, student *t* test. **e** The calculated combination index (CI) values of the (**c**, **d**) were shown. The meaning of CI was interpreted as: CI > 1, antagonistic effect; CI = 1, additive effect; and CI <  1, synergistic effect

To examine the role of DNMT3b in sorafenib resistance in HCC, the sorafenib resistant Hep3B cells were generated in medium with increasing of sorafenib concentration stepwisely. When compared with the naïve cells, the sorafenib-resistant HCC cells showed a higher value of IC_50_ (15.76 μM vs. 9.52 μM) (Fig. 6a), and expressed higher levels of *IL-6R*, *DNMT3b* and stemness-related genes (Fig. 6b and Additional file 1: Figure S7). Inhibition of DNMT3b by nanaomycin A significantly increased the sorafenib sensitivity in a dose-dependent manner (Fig. 6c and d). The similar effect of nanaomycin A on lipo-DOX sensitivity was also shown (Additional file 1: Figure S8). The synergistic effect of nanaomycin A and sorafenib on the suppression of sorafenib-resistant Hep3B proliferation were shown in Fig. 6e and Table 2. These results demonstrate that targeting DNMT3b with nanaomycin A showed a synergistic effect with sorafenib in the treatment of sorafenib resistant HCC. The results revealed that DNMT3b was involved in the IL-6-induced OCT4 expression axis in sorafenib-resistant Hep3B cells.

**Table 2:** IC50 of Sorafenib and nanaomycin in Hep3B naïve cells and Hep3Bresistant cells

Drugs (μM)	Inhibitory concentration, IC50 (μM)	
	Hep3B naive cells	Hep3B resistant cells
Sorafenib	9.56 ± 1.77	15.7 ± 3.11
Nanaomycin	5.56 ± 3.19	7.48 ± 5.92
Sorafenib + Nanaomycin 2	6.38 ± 0.81	7.71 ± 0.88
Sorafenib + Nanaomycin 5	3.01 ± 0.75	4.68 ± 0.95
Sorafenib + Nanaomycin 10	1.42 ± 0.20	2.06 ± 0.43

## Discussion

The inflammatory cytokine IL-6 is known to be involved in the pathogenesis and progression of various cancers. IL-6 has been shown to increase cancer stemness-related genes and properties [14, 17] by up-regulating DNMTs [43, 44, 48–50] or down regulating DNMTs [51]. However, the complicated interplay among IL-6, DNMTs, and cancer stemness-related genes, such as OCT4, still remains unclear. In this study, we demonstrated that the niche inflammatory cytokine IL-6 increased OCT4 expression via DNMT3b, in a STAT3-dependent manner in HCC. We found that IL-6 levels and OCT4/DNMT3b expression were positively correlated with early tumor recurrence in HCC patients. The expression of DNMT3b/OCT4 may confer sorafenib resistance in HCC, and the DNMT3b inhibitor exhibited a synergistic effect with sorafenib on sorafenib-resistant liver cancer. Findings of this study suggest a therapeutic strategy for inhibiting DNMT3b to enhance the sorafenib sensitivity in HCC cells.

The pluripotent transcription factor OCT4, which is essential for the self-renewal and maintenance of embryonic pluripotent stem cells, plays an important role in the initiation and progression of malignant diseases [14, 20, 24–28, 30, 52]. In HCC, OCT4 expression may lead to tumor recurrence and chemotherapy resistance [14, 22, 26, 52, 53]. DNA methylation of both CpG and non-CpG island promoters has been associated with the regulation of OCT4 expression in embryonic stem cells and trophoblast stem cells [21]. In somatic cancers, aberrant DNMT regulation can drive the production of cancer stem-like phenotypes through OCT4 reprogramming in glioblastoma [30]. *OCT4* expression was shown to correlate with *DNMT1* and *DNMT3b* expression in primary glioblastoma neurosphere, and the transgenic *OCT4/SOX2* co-expression is able to increase the expressions of *DNMTs* in gliomas [30]. Recently, a study on human HCC tissues also demonstrated that expression of DNMT1 and DNMT3b contributes to hepatocellular carcinogenesis [33]. The number of methylated genes and the mRNA levels of *DNMT1*, *DNMT3a* and *DNMT3b* were shown to be increased progressively from normal liver, chronic hepatitis/cirrhosis to HCC [34].

As for the niche factors on DNMT expression in HCC, recent studies have suggested that both HBV and HCV upregulate DNMTs in HCCs associated with poor outcomes [14, 38]. Consistent with this, our results showed that, when compared to patients with non-HB(C)V-HCC (NBNC), patients with HBV-HCC or HCV-HCC have higher expression levels of *DNMT3b* and *DNMT1* (Additional file 1: Figure S9). Infection with HBV or HCV has been known to induce chronic systemic inflammation [54]. Patients with HBV-HCC have high serum IL-6 levels [14]. Our previous study demonstrated that HBV-derived niche IL-6 can upregulate OCT4 expression through insulin-like growth factor 1 receptor (IGF-1R) signaling and the OCT4 expression can result in early tumor recurrence [14].

We have previously demonstrated the positive correlation of another pluripotent transcription factor, NANOG, with IGF-1R activation (phospho-IGF-1R) in HCC tissues, and showed that IGF-1/IGF-1R activation regulates NANOG expression in HCC in vitro and in vivo [14]. In addition, the IGF-1R signaling was demonstrated to regulate NANOG, which controls CSC self-renewal and maintains CSC-related properties in HCC [55]. In this study, we found that *DNMT3b* was positively correlated with *NANOG* (*R* = 0.7330, *P* < 0.0001) and *IGF-1R* (*R* = 0.5419, *P* < 0.0001) in HCC tissues (Additional file 1: Figure S10a and b). No significant correlation between *DNMT1* and *NANOG or IGF-1R* was observed (Additional file 1: Figure S10c and d).

**Fig. 7:**
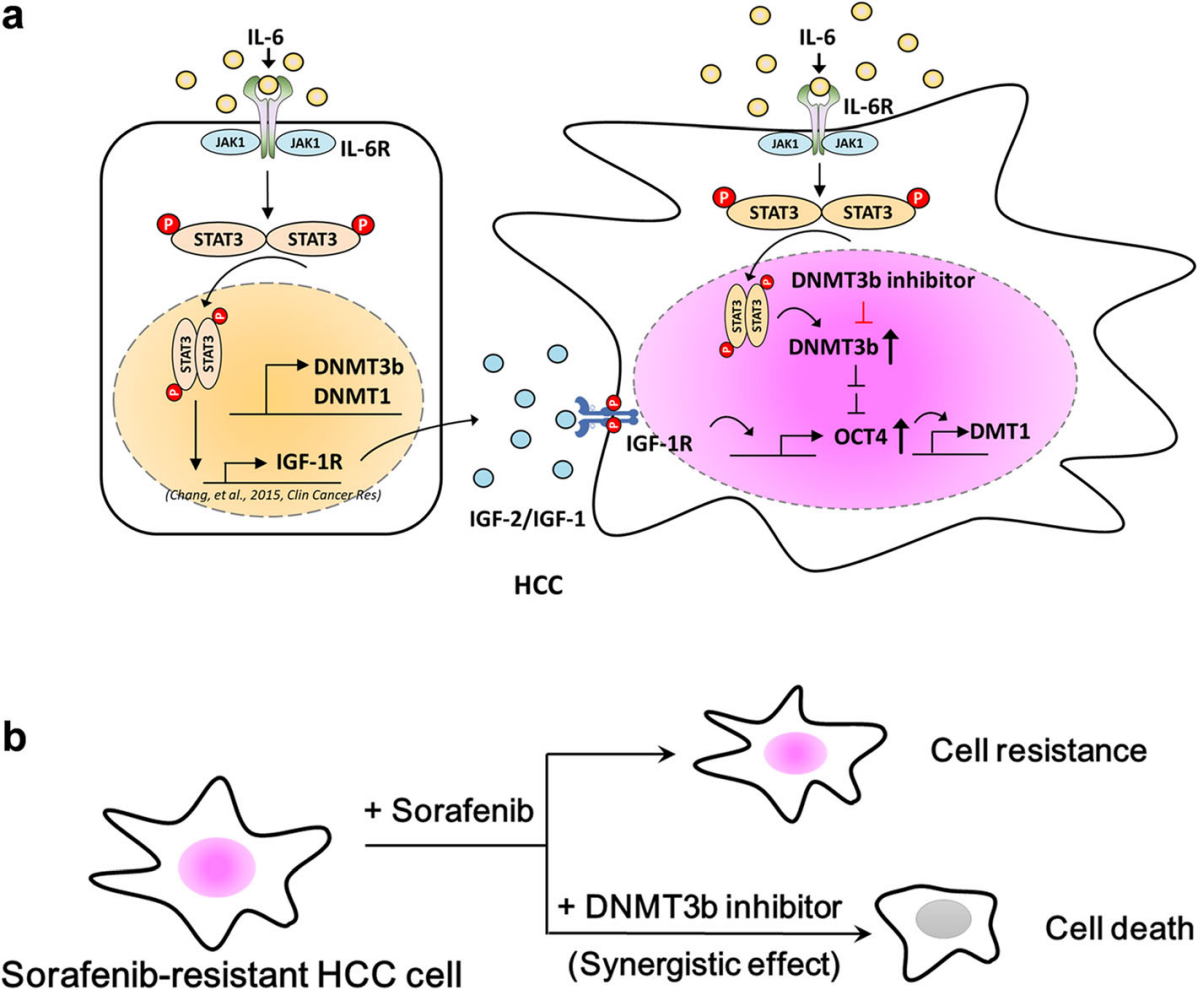
IL-6 increases the expression of OCT4 through DNMT3b and IGF-1R activation in human HCC. **a** Model of how IL-6 increases the expression of OCT4 through p-STAT3-DNMT3b-OCT4-DNMT1 activation in human HCC. **b** The combination use of nanaomycin and sorafenib synergistically suppresses the cell proliferation of sorafenib resistant HCC cells

Constitutively active STAT3 signaling has been documented in human cancers including HCC and oral cancer [17, 42]. Factors involving inflammatory cytokines, growth factors and virus infection have been shown to activate hepatic STAT3 signaling [14, 17]. However, the roles of IL-6, DNMTs, and OCT4 in HCC still remain unclear. In oral cancer, a role for an IL-6-DNMT3b axis in cell proliferation and epithelial-mesenchymal transition (EMT) and poor cancer prognosis was observed [42]. But, the relationship between IL-6-DNMT3b and OCT4 expression was not discussed in this literature [42]. Liu et al. showed that IL-6 enriched lung cancer stem-like cell populations by the inhibition of cell cycle regulators via DNMT1 upregulation [48]. However, the role of IL-6 in the regulation of DNMT3b was not addressed. Recently, Quan et al. suggested that IL-6 can induce cell proliferation via STAT3 dependent upregulation of DNMT1 and DNMT3b in renal cell carcinoma [50]. However, OCT4 was not examined in that study [50]. Advancing these previous findings and extending our previous study, which showed that IL-6/STAT-3 upregulates OCT4 through IGF-1R [14], the current work demonstrates that the IL-6-STAT3 signaling drives OCT4 expression through the regulation of DNMT3b in HCC (Fig. 7).

IL-6, DNMTs and cancer stemness-related genes like OCT4 usually interplay with one another to promote carcinogenesis. Kaplan-Meier analysis of our patients showed that *DNMT3b* and *OCT4* gene expression levels were positively correlated with tumor recurrence (Fig. 1g). HCC is well known for its marked genetic heterogeneity, which remains a major obstacle in attempts to develop an effective therapy [56]. The IL-6-STAT3 mediated IGF-1/IGF-1R signaling or DNMT3b-OCT4-DNMT1 regulation in HCC may be two of the multiple factors involved in stemness expression and tumor recurrence. These observations strongly suggest that the overall outcome of HCC may depend not only on blocking the IGF-1R/OCT4 pathways, but also other complementary and/or synergistic factors like IL-6 and DNMT3b.

The promoter region of the OCT4 gene is typically hypermethylated in somatic cells such as those in the liver [21], thus it is likely that there are other mediators between DNMT3b and OCT4 not yet delineated in our study. It is well known that microRNAs (miRNAs) are involved in diverse biological processes, such as cell proliferation, tumorigenesis, apoptosis, invasion, and angiogenesis of cancer cells [57]. miRNAs are important for the regulation of cancer stem-like properites [58]. A previous study revealed that miR-335 negatively regulates osteosarcoma stem-like properites [59]. Further, stem cell-related genes *OCT4* and *SOX2* are among the target genes regulated by miR-145, suggesting that miR-145 may play an important role in the maintenance of cancer stem cells [60]. Moreover, miR-145 was shown to play an oncogenic role in hepatocarcinogenesis [22]. Through software predictions and with support from previous studies, we propose that miR-145 may regulate OCT4 expression through DNMT3b.

Sorafenib is the first FDA-approved targeted therapy for patients with advanced HCC. However, the sorafenib has a number of disabling side effects [61], and its efficacy has not been satisfied. Currently many attempts in combining with other agents to minimize the sorafenib dose have been reported, including the epigenetic therapeutics [62]. Liu et al., showed that DNMT1 was upregulated through STAT3 signaling pathway in the sorafenib-resistant non HBV/HCV-infected HepG2 and Huh7 cells. Blocking DNMT1 by their inhibitor can decrease colony formation and enhanced sorafenib sensivity in HCC cells resistant to sorafenib [63]. Besides, in hormone refractory prostate cancers, the tumor cells presented drug resistance and increased the expressions of DNMT1 and DNMT3b. Down-regulating of the DNMT1 and DNMT3b activity by DNMT inhibitor acacytidine results in the increase of the drug sensitivity in cells [64].

Different from the Liu’s study in HCC, our results demonstrated that the combination of sorafenib and the low dose nanaomycin A (2 μM), which specific inhibit the DNMT3b can synergistically suppressed the proliferation of sorafenib-resistant HBV^+^Hep3B cells (Fig. 6de). The fact that low dose nanaomycin A did not suppress DNMT1 expression in Hep3B cells (Fig. 5a), indicates that the role of DNMT3b is specific in HBV^+^Hep3B cells, and the differential DNMT expression patterns in HCCs of different etiologies would be the critical factors. Our results demonstrated that targrting DNMT3b would increase the sorafenib sensitivity and augment the therapeutic effect of sorafenib on sorafenib-resistant HCC cells, in particular for the HBV-HCC.

## Conclusions

In summary, the current study demonstrated that the DNMT3b/OCT4 expression confers sorafenib resistance and poor prognosis of HCC through IL-6/p-STAT-3 regulation. The mechanism of IL-6/p-STAT-3 signaling in the sequential activation signaling of the DNMT3b-OCT4-DNMT1 axis in HCC was successfully delinated. Targeting DNMT3b showed a synergistic effect with sorafenib in the treatment of the sorafenib resistant HCCs (Fig. 7). The results of this study could provide therapeutic strategies for the HCC patients expressing cancer stemness properties such as DNMT3b/OCT4 expression and/or sorafenib resistance.

## Additional file

**Additional file 1.** Supplementary information.

## Data Availability

All the data related to the study are included within the article and the supplemental material.

## Abbreviations

CSC: Cancer stem cell
DNMT: DNA methyltransferase
HBV: Hepatitis B virus
HCC: Hepatocellular carcinoma
IL-6: Interleukin-6
Lipo-DOX: Liposomal doxorubicin
OCT4: Octamer-binding transcription factor4
STAT: Signal transducer and activator of transcription
TCGA: The Cancer Genome Atlas
