# Correction to: DNMT3b/OCT4 expression confers sorafenib resistance and poor prognosis of hepatocellular carcinoma through IL-6/STAT3 regulation

**DOI:** 10.1186/s13046-019-1518-z

**Published:** 2020-01-13

**Authors:** Ssu-Chuan Lai, Yu-Ting Su, Ching-Chi Chi, Yung-Che Kuo, Kam-Fai Lee, Yu-Chih Wu, Pei-Chi Lan, Muh-Hwa Yang, Te-Sheng Chang, Yen-Hua Huang

**Affiliations:** 1 Graduate Institute of Medical Sciences, College of Medicine, Taipei Medical University, Taipei, 11031 Taiwan; 2 Department of Biochemistry and Molecular Cell Biology, School of Medicine, College of Medicine, Taipei Medical University, Taipei, 11031 Taiwan; 3 TMU Research Center for Cell Therapy and Regeneration Medicine, Taipei Medical University, Taipei, 11031 Taiwan; 4 Department of Dermatology, Chang Gung Memorial Hospital, Linkou, Taoyuan, 33305 Taiwan; 5 College of Medicine, Chang Gung University, Taoyuan, 33302 Taiwan; 6 Department of Pathology, Chang Gung Memorial Hospital, Chiayi, 61363 Taiwan; 7 School of Respiratory Therapy, College of Medicine, Taipei Medical University, Taipei, 11031 Taiwan; 8 Institute of Clinical Medicine, College of Medicine, National Yang Ming University, Taipei, 11221 Taiwan; 9 Division of Medical Oncology, Taipei Veterans General Hospital, Taipei, 11217 Taiwan; 10 School of Traditional Chinese Medicine, College of Medicine, Chang Gung University, Taoyuan, 33382 Taiwan; 11 Division of Internal Medicine, Department of Gastroenterology and Hepatology, Chang Gung Memorial Hospital, Chiayi, 61363 Taiwan; 12 International PhD Program for Cell Therapy and Regeneration Medicine, College of Medicine, Taipei Medical University, Taipei, 11031 Taiwan; 13 Center for Reproductive Medicine, Taipei Medical University Hospital, Taipei Medical University, Taipei, 11031 Taiwan; 14 Ph.D. Program for Translational Medicine, College of Medical Science and Technology, Taipei Medical University, Taipei, 11031 Taiwan; 15 Comprehensive Cancer Center of Taipei Medical University, Taipei, 11031 Taiwan

**Correction to: J Exp Clin Cancer Res (2019) 38:474**

**https://doi.org/10.1186/s13046-019-1442-2**

In the original publication of this article [1], labelling within Fig. 7a was incorrect. The updated figure is shown below, with ‘DMT1’ now corrected to read ‘DNMT1’.

**Fig. 7:**
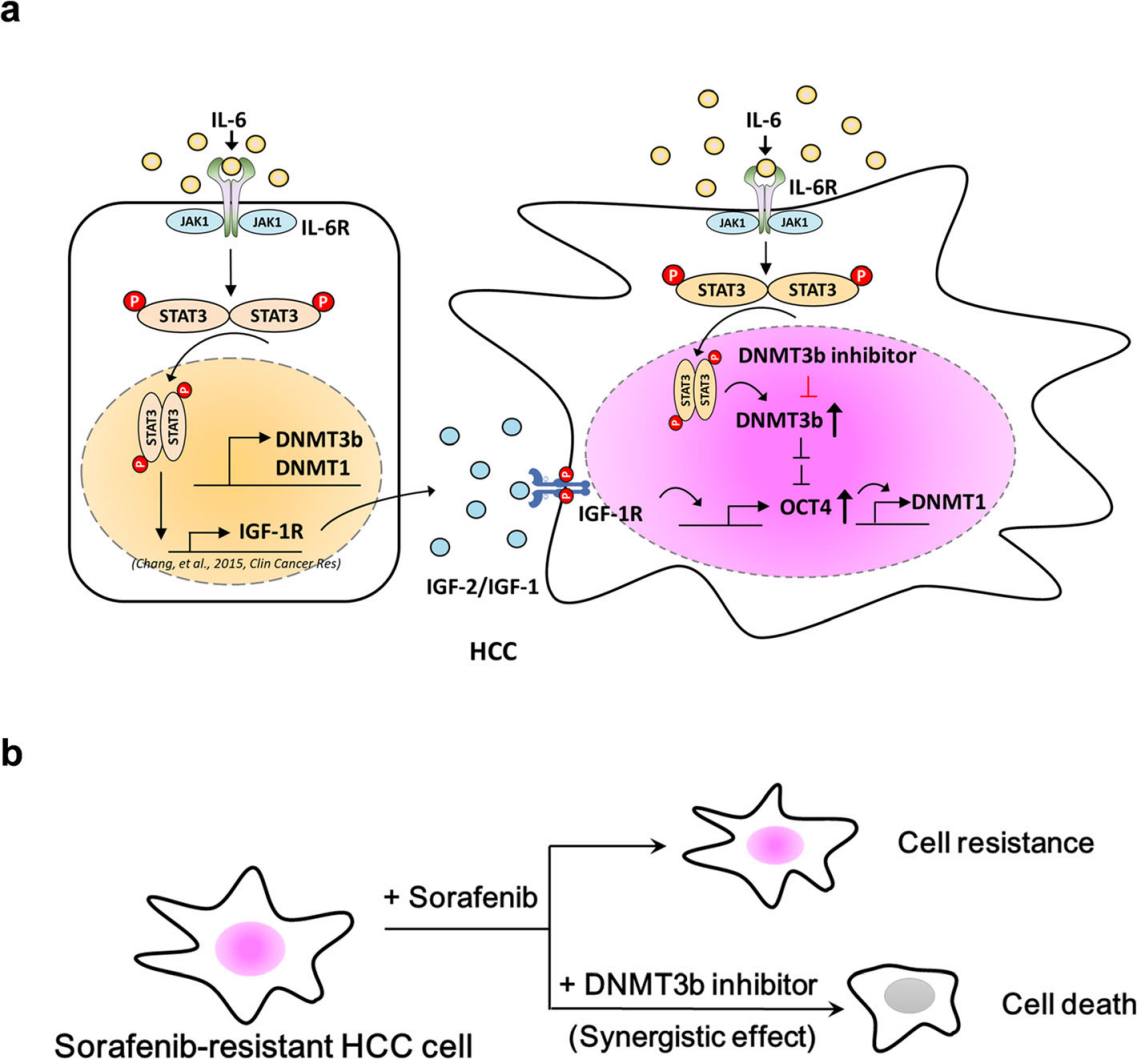
IL-6 increases the expression of OCT4 through DNMT3b and IGF-1R activation in human HCC. **a** Model of how IL-6 increases theexpression of OCT4 through p-STAT3-DNMT3b-OCT4-DNMT1 activation in human HCC. **b** The combination use of nanaomycin and sorafenibsynergistically suppresses the cell proliferation of sorafenib resistant HCC cells

The authors sincerely apologize for the inconvenience caused to the readers.
