# Supplementary material for: DNMT3b/OCT4 expression confers sorafenib resistance and poor prognosis of hepatocellular carcinoma through IL-6/STAT3 regulation
Source: J Exp Clin Cancer Res. 2019 Nov 26;38:474. doi: 10.1186/s13046-019-1442-2 (PMC6878666; doi:10.1186/s13046-019-1442-2)
Supplement: Supplementary file 1 — Additional file 1. Supplementary information. [file 13046_2019_1442_MOESM1_ESM.docx]

**Additional file 1**

**
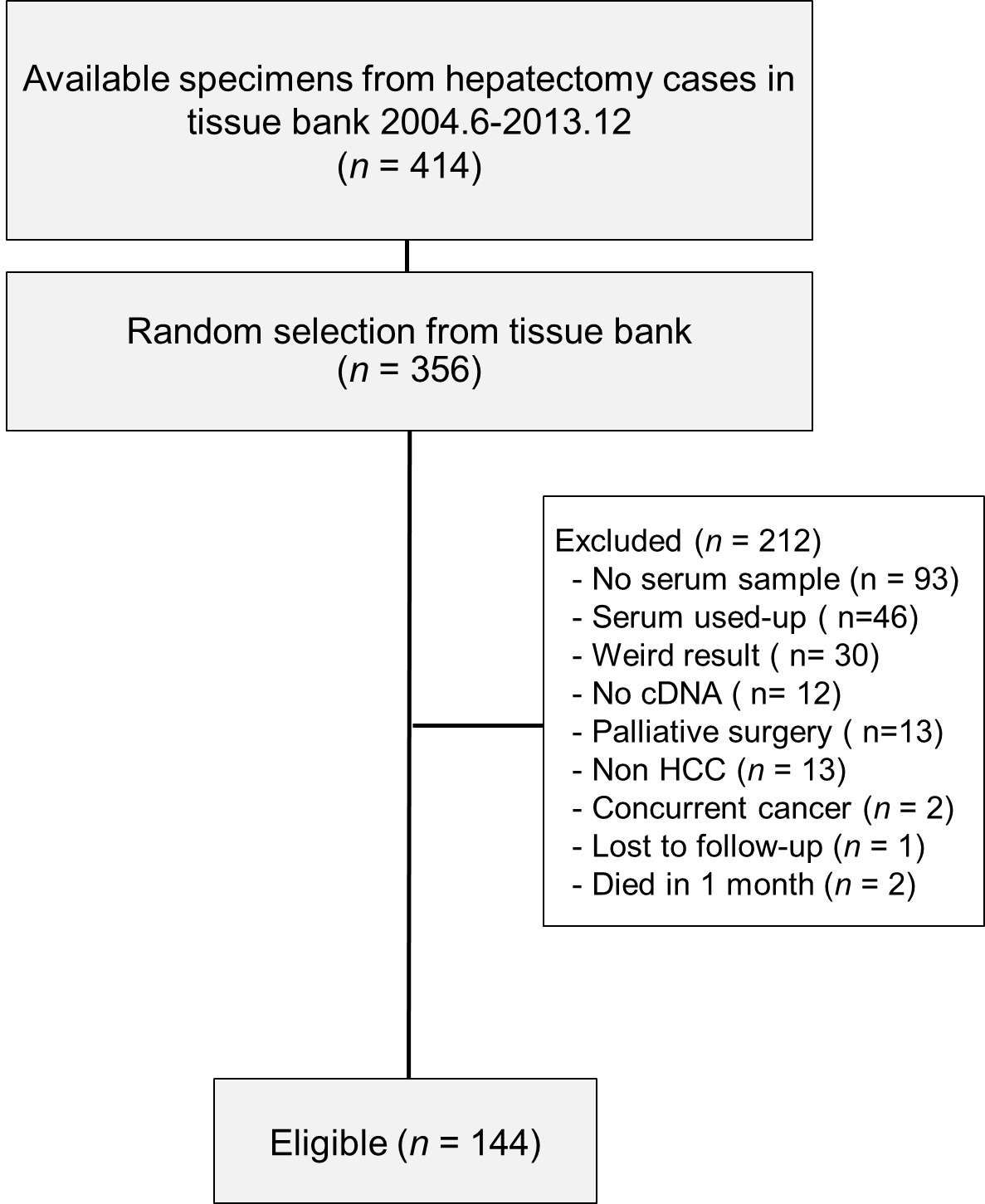
**

**Figure S1. Flow diagram of all patients with HCC enrolled in this study**

Pairs of liver tumor and peri-tumor specimens from 414 patients receiving hepatectomy between 2004.6 and 2013.12 were available in the tissue bank. 356 samples were randomly selected for our study. Of these, 212 were excluded for a variety of reasons, including no serum sample (*n* = 93), serum used-up (*n* = 46), atypical result (*n* = 30), no cDNA (*n* = 12) , palliative surgery (*n* = 13) , non HCC (*n* = 13) , concurrent cancer (*n* = 2) , lost to follow-up (*n* = 1) and died in one month (*n* = 2). The HCC specimens from the 144 eligible patients were subjected to quantitative real-time PCR.


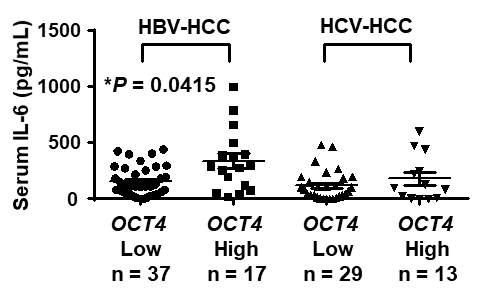


**Figure S2. Correlation of the expression of serum IL-6 and *OCT4* genes in human HBV-HCC and HCV-HCC**

The differences in serum levels of IL-6 between patients with low *OCT*4 expression (T/PT < 2-fold; *n* = 37) and high *OCT4* expression (T/PT > 2-fold; *n* = 17) with HBV-HCC by the Mann–Whitney *U* test. The differences in serum levels of IL-6 between patients with low *OCT*4 expression (T/PT < 2-fold; *n* = 29) and high *OCT4* expression (T/PT > 2-fold; *n* = 13) with HCV-HCC by the Mann–Whitney U test.


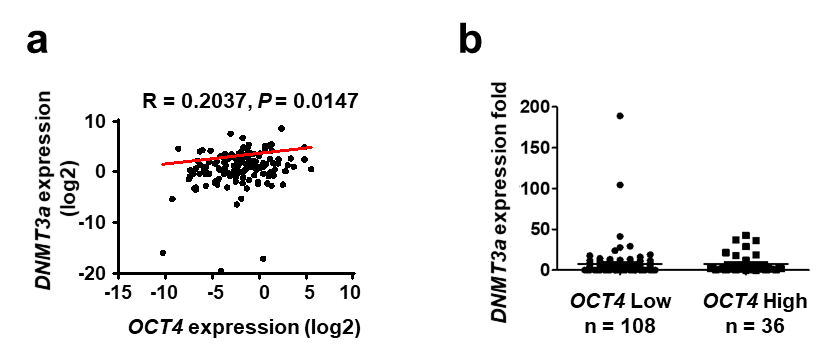


**Figure S3. Correlation of the expression of *DNMT3a* and *OCT4* genes in human HCC**

**(a)** Correlations between expression levels of *OCT4* with *DNMT3a* (*R* = 0.2037, *P* = 0.0147) by Spearman correlation analysis. **(b)** The differences in *DNMT3a* between HCC patients with low *OCT*4 expression (T/PT < 2-fold; *n* = 108) and high *OCT4* expression (T/PT > 2-fold; *n* = 36) by the Mann–Whitney *U* test.

**
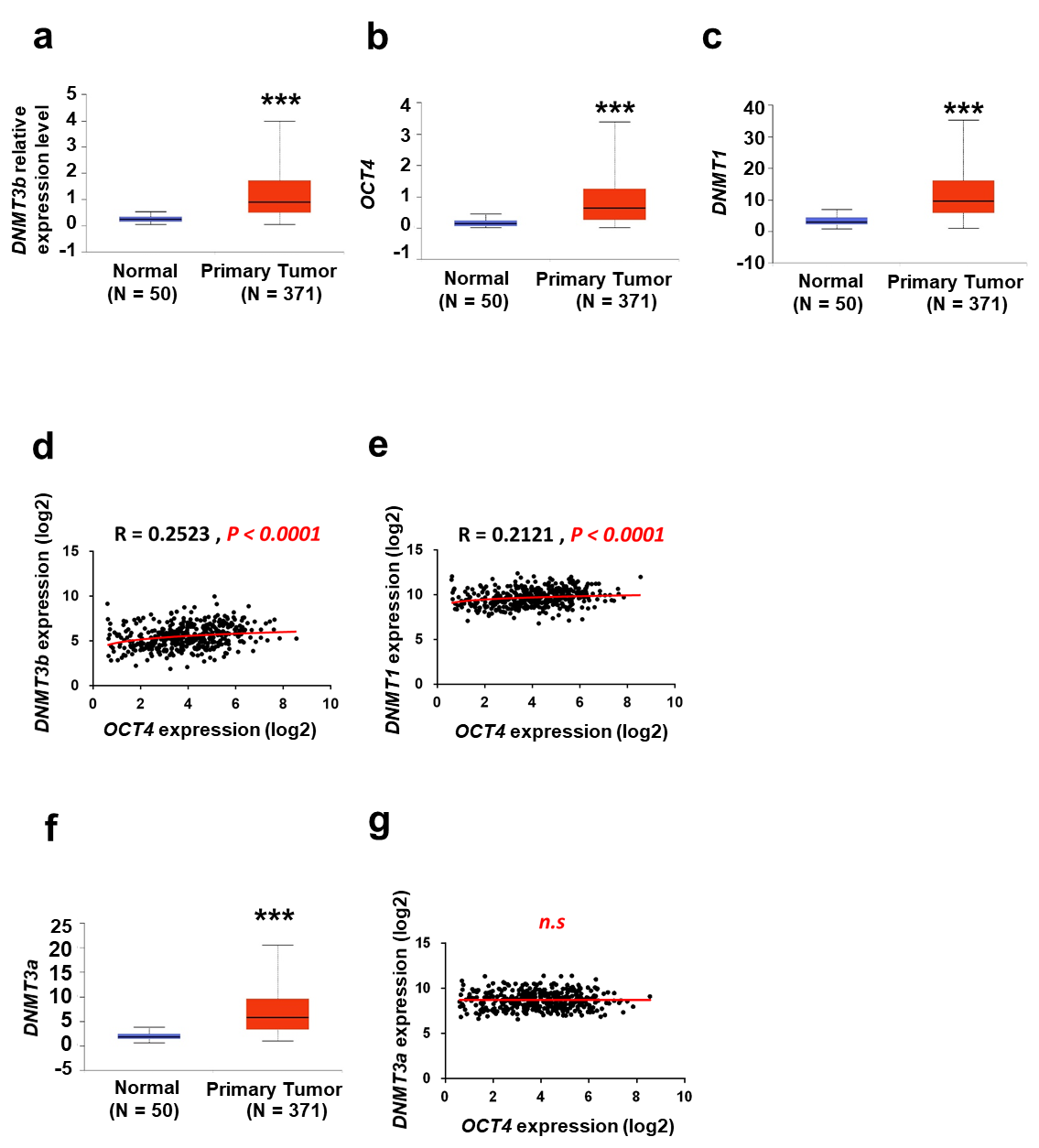
**

**Figure S4. Correlation of the expression of *DNMTs* and *OCT4* genes in human HCC in TCGA**

**(a, b, c)** Boxplot showing relative gene expression of *DNMT3b/OCT4/DNMT1* in normal versus primary tumor samples by Student *t* test. **(d, e)** Correlations among transcriptional levels of *DNMT3b, DNMT1 and OCT4* in liver tumor samples (T) and adjacent normal liver tissues (N) were analyzed using real-time qRT-PCR and Spearman correlation analysis. *P* < 0.0001. **(f)** Comparisons between quantitative protein expression levels in T and N tissues in *DNMT3a* and in paired samples (N and T tissues from the same patient) by Student *t* test. **(g)** Correlations between expression levels of *OCT4* with *DNMT3a* (*n.s.,* no significance) in HCC tissues (*n* = 144) by Spearman correlation analysis.


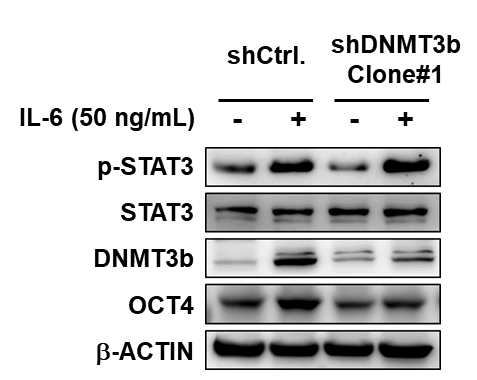


**Figure S5. DNMT3b knockdown by transient treansfection with shRNA reduced the OCT4 expression in sorafenib-resistant Hep3B cells.**

Forty-eight hours after transfection of sorafenib-resistant Hep3B cells with DNMT3b shCtrl or shDNMT3b clone#1, the cells were treated with IL-6 for 24 h. The protein levels of DNMT3b and OCT4 were evaluated using Western blotting.

**Figure S6. Forced OCT4 expression increased cell viability in Hep3B cells.**

IC_50_ values of sorafenib in Hep3B cell lines were determined using the water-soluble tetrazolium salt (WST) assay. The cell viabilities of Hep3B cells and forced OCT4 expressing Hep3B cells with sorafenib treatment (0, 1, 2, 5, 10, 15, and 20 μM) were determined. The cell viabilities of Hep3B control and overexpressing OCT4 cells were measured after 48 h of treatment with the indicated sorafenib concentrations.


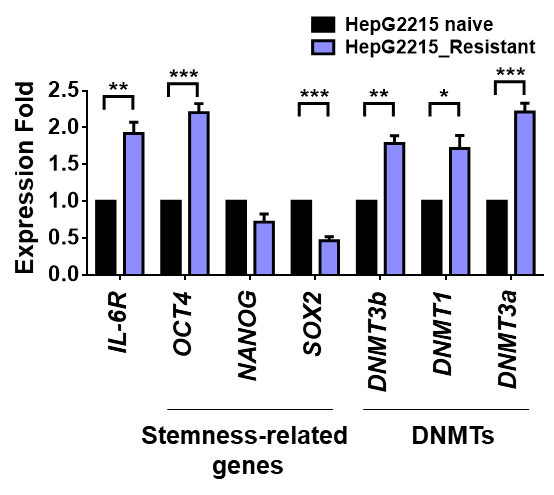


**Figure S7. Comparative gene expression profiling of sorafenib naïve or resistant HepG2215 cells**

The mRNA expression levels of the indicated *IL-6R*, stemness-related genes and *DNMT*s were quantified by real-time RT-qPCR**.** **P* <0.05, ***P* <0.01, ****P* < 0.001, student *t* test.

**
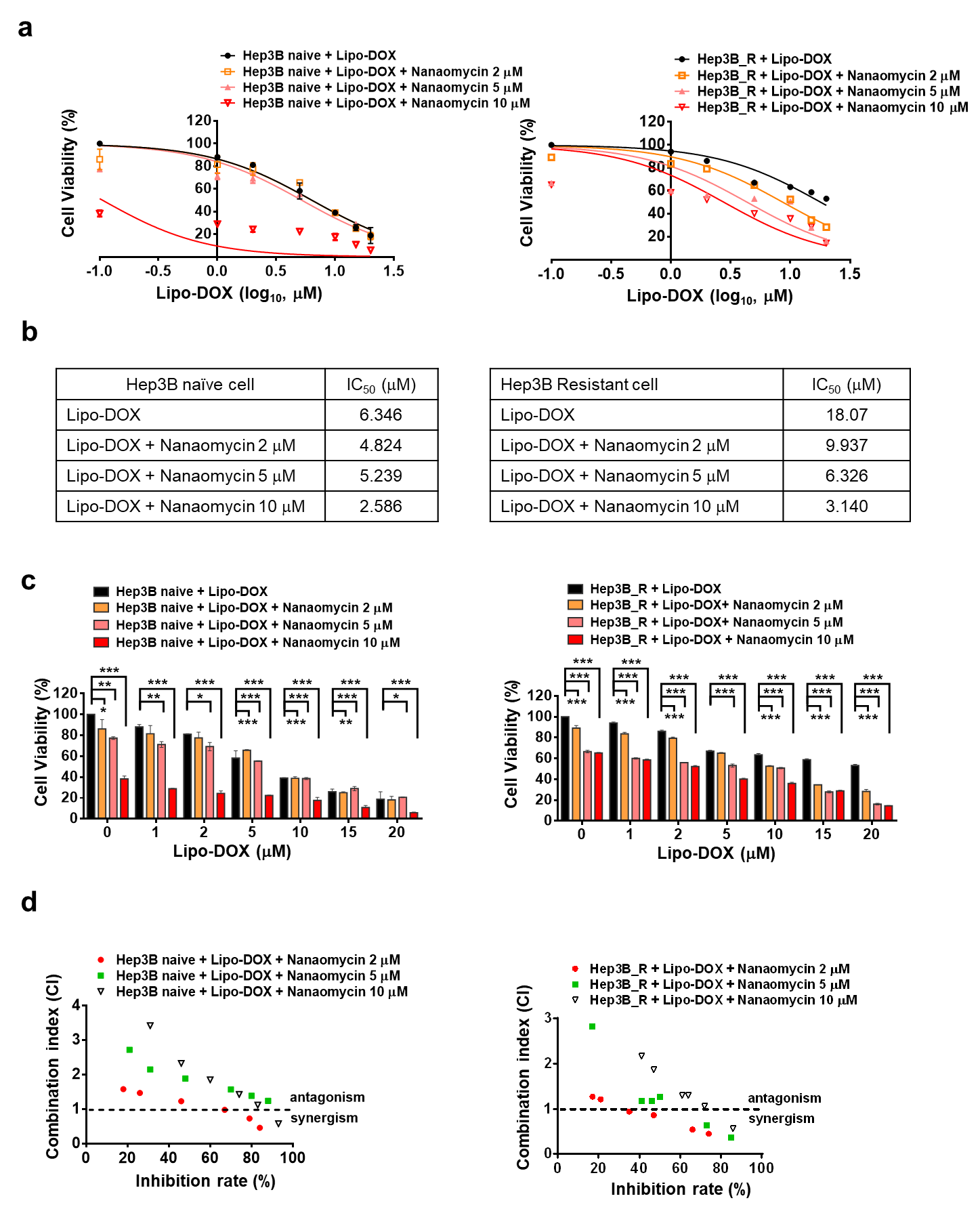
**

**Figure S8. Concentration-effect curves of single-agent Lipo-doxorubicin (Lipo-DOX) and nanaomycin combinational used in Hep3B sorafenib naïve/reisitant cells**

**(a)** The combinational usage of lipo-DOX and nanaomycin A (2, 5, and 10 μM) for 48h in Hep3B naïve/resistant cells. Cell viabilities were determined by WST assay. **(b)** IC_50_ values were calculated using GraphPad Prism 6 software. **(c)** The combination of different dosage of lipo-DOX and nanaomycin A (2, 5, and 10 μM) on HCC naïve/resistant. Cells were treated with sorafenib alone, or in combination at the indicated concentrations of nanaomycin A for 48h. Cell viabilities were determined by WST assay. **P* <0.05, ***P* <0.01, ****P* < 0.001, student *t* test. **(d)** Calculated combination index (CI) values of sorafenib combination with nanaomycin A in HCC naïve/resistant cells. The meaning of CI interpreted as: CI > 1, antagonistic effect; CI = 1, additive effect; and CI < 1, synergistic effect.


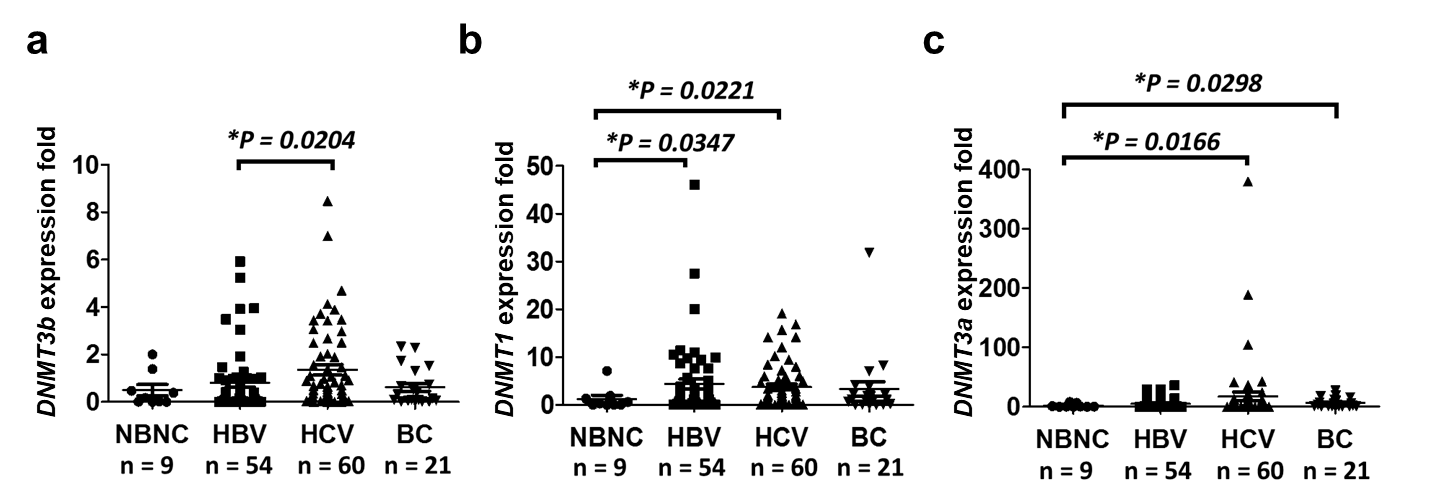


**Figure S9. Correlation of the expression of *DNMTs* and various viral etiologies in human HCC**

Gene levels of *DNMTs* in patients with HCC are presented according to the various viral etiologies (*n* = 144) including NBNC (Neither HBV nor HCV) (*n* = 9), HBV (*n* = 54), HCV (*n* = 60), or BC (HBV and HCV) (*n* = 21). By the Mann–Whitney *U* test.


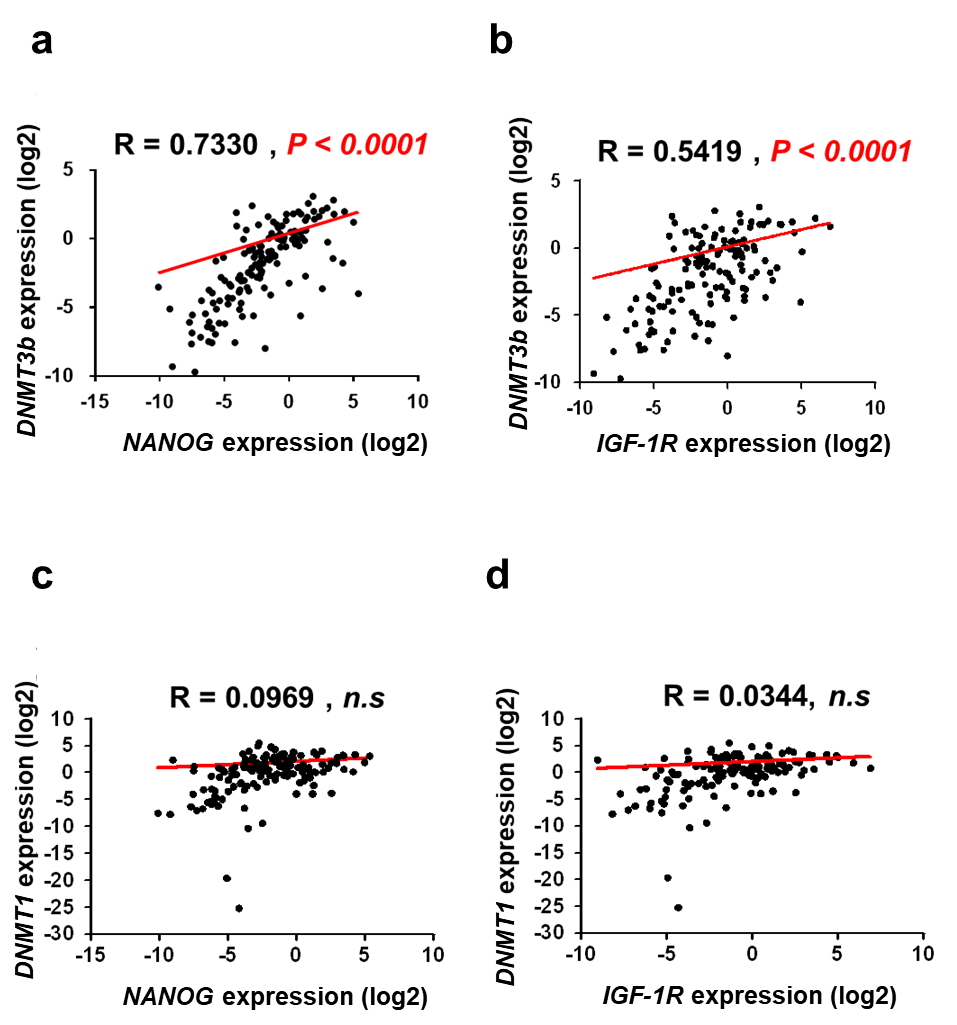


**Figure S10. Correlation of the expression of *DNMT3b/DNMT*1 with the *NANOG/IGF-1R* genes of human HCC**

**(a)** Positive correlations between expression levels of *NANOG* with *DNMT3b* (*R* = 0.7330, *P* <0.0001) and *IGF-1R* with *DNMT3b* (*R* = 0.5419, *P* <0.0001) **(b)**. **(c, d)** No correlation between expression levels of *Nanog/IGF1-R* with *DNMT1* in HCC tissues was observed by Spearman correlation analysis (*n* = 144).


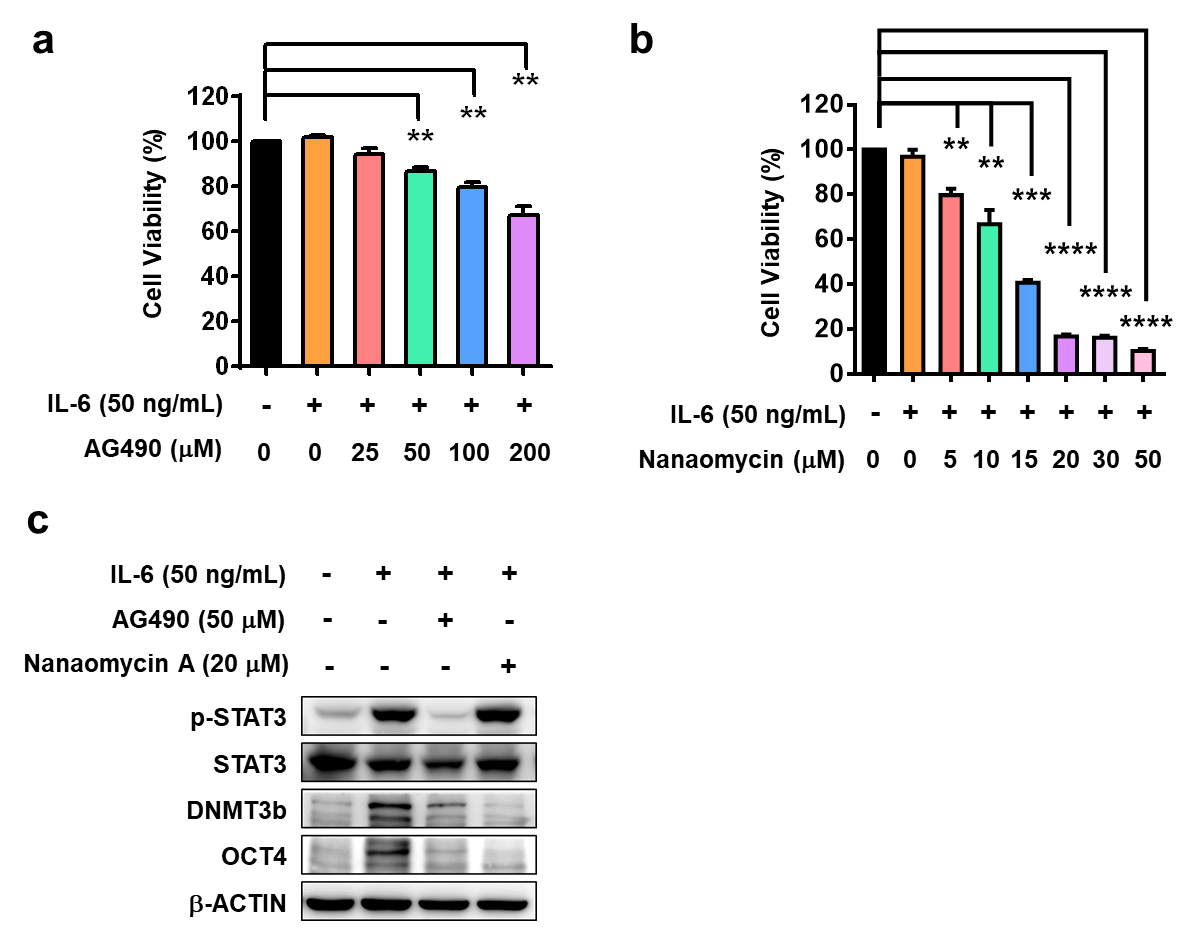


**Figure S11. p-STAT3/DNMT3b signaling regulates OCT4 expression in sorafenib-resistant human Hep3B cells (For reviewer only)**

**(a and b)** IL-6 in combination with AG490/nanaomycin suppresses sorafenib-resistant Hep3B cell proliferation. Sorafenib-resistant Hep3B cells were treated with IL-6, AG490, and nanaomycin at the indicated doses. After 48 h of treatment, the cell viability was assessed using the water soluble tetrazolium salt (WST) assay. ***P* < 0.01, ****P* < 0.001, *****P* < 0.0001, indicate significant differences between the treatment and control groups. **(c)** Western blot analysis of p-STAT3/DNMT3b/OCT4 proteins in sorafenib-resistant Hep3B cells treated with IL-6 (50 ng/mL), AG490 (50 μM), and nanaomycin A (20 μM), or the combination IL-6 and nanaomycin.

**
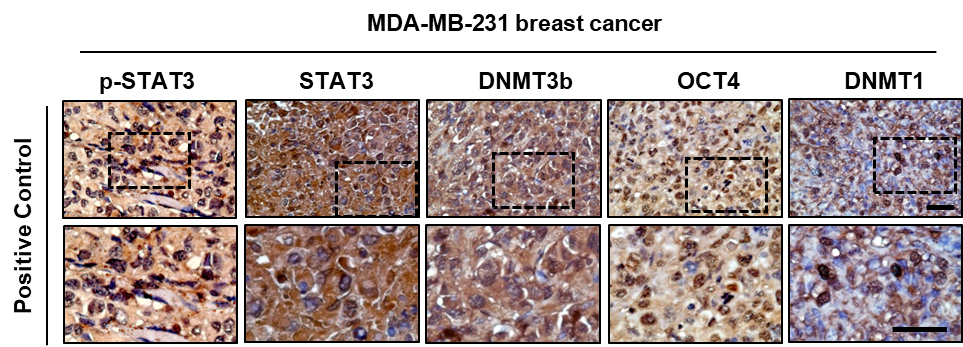
**

**Figure S12. Immunohistochemical expression of p-STAT3, DNMT3b, OCT4, and DNMT1 in MDA-MB-231 breast cancer cells (For reviewer only)**

Positive control, MDA-MB-231 breast cancer cell line. Scale bar, 100 μM.

Table S1. Real-time PCR primers and product size

| **Gene** | **Accession No.** | **Primer sequences** | **Product Size (bp)** |
| --- | --- | --- | --- |
| **DNMT3b** | NM_006892 | F:5’-TTGGAATAGGGGACCTCGTGTG-3’  R:5’-AGAGACCTCGGAGAACTTGCCA-3' | 150 |
| **DNMT1** | NM_001318731 | F:5’-CGACTACAAAGGCAGCAACC-3’  R:5’-TGGAGTGGACTTGTGGGTGTTCT-3’ | 134 |
| **DNMT3a** | NM_153759 | F:5’-CGAGTCCAACCCTGTGATGATTG-3’  R:5’-GCTGGTCTTTGCCCTGCTTTATG-3’ | 220 |
| **OCT4** | NM_002701 | F:5’-CAACTCCGATGGGGCCT-3’  R:5’-CTTCAGGAGCTTGGCAAATTG-3’ | 148 |
| **NANOG** | NM_024865 | F:5’-CCTGTGATTTGTGGGCCTG-3’  R:5’-GACAGTCTCCGTGTGAGGCAT-3’ | 114 |
| **SOX2** | NM_003106 | F:5’-GTATCAGGAGTTGTCAAGGCAGAG-3’  R:5’-TCCTAGTCTTAAAGAGGCAGCAAA-3’ | 77 |
| **IL-6R** | NM_000565 | F:5’- AGACAAGCCCAGCAATGAAAA-3’  R:5’- GCGAAAGGATGAAAGTGACCAT-3’ | 97 |
| **IGF1-R** | NM_000875.3 | F:5’-CTCCTGTTTCTCTCCGCCG-3’  R:5’-ATAGTCGTTGCGGATGTCGAT-3’ | 85 |
| **β-2M** | NM_004048 | F:5’-GTCTCGCTCCGTGGCCTTA-3’  R:5’-TGAATCTTTGGAGTACGCTGGATA-3’ | 80 |

Table S2. List of antibodies

| **Protein** | **Assay** | **Cat. No.** | **Company** | **Origin** | **Dilution** | **Incubation Period** |
| --- | --- | --- | --- | --- | --- | --- |
| **DNMT3b** | WB | ab13604 | abcam | Mouse | 1:1000 | Overnight,4°C |
| **DNMT1** | WB | ab13537 | abcam | Mouse | 1:1000 | Overnight,4°C |
| **DNMT3a** | WB | ab2850 | abcam | Rabbit | 1:1000 | Overnight,4°C |
| **OCT4** | WB | sc-5279 | Santa Cruz | Mouse | 1:500 | Overnight,4°C |
| **Flag** | WB | F1804 | Sigma | Mouse | 1:3000 | Overnight, 4°C |
| **p-STAT3** | WB | #9145 | Cell Signaling | Rabbit | 1:2000 | Overnight,4°C |
| **STAT3** | WB | sc-8019 | Santa Cruz | Mouse | 1:1000 | Overnight,4°C |
| **N-cadherin** | WB | Ab76011 | abcam | Rabbit | 1:2000 | Overnight,4°C |
| **β-actin** | WB | sc-47778 | Santa Cruz | Mouse | 1:1000 | Overnight,4°C |
| **DNMT3b** | IHC | ab13604 | abcam | Mouse | 1:250 | Overnight,4°C |
| **DNMT1** | IHC | ab13537 | abcam | Mouse | 1:250 | Overnight,4°C |
| **DNMT3a** | IHC | ab2850 | abcam | Rabbit | 1:250 | Overnight,4°C |
| **OCT4** | IHC | sc-5279 | Santa Cruz | Mouse | 1:250 | Overnight,4°C |
| **Control IgG** | IHC | #76780 | JacksonImmuno  Research | Mouse | 1:250 | Overnight,4°C |
